# Genome-Wide Variation in Potyviruses

**DOI:** 10.3389/fpls.2019.01439

**Published:** 2019-11-12

**Authors:** Deepti Nigam, Katherine LaTourrette, Pedro F. N. Souza, Hernan Garcia-Ruiz

**Affiliations:** Department of Plant Pathology, Nebraska Center for Virology, University of Nebraska-Lincoln, Lincoln, NE, United States

**Keywords:** potyvirus, SNP, SAP, diversity, adaption, host range

## Abstract

Potyviruses (family *Potyviridae*, genus *Potyvirus*) are the result of an initial radiation event that occurred 6,600 years ago. The genus currently consists of 167 species that infect monocots or dicots, including domesticated and wild plants. Potyviruses are transmitted in a non-persistent way by more than 200 species of aphids. As indicated by their wide host range, worldwide distribution, and diversity of their vectors, potyviruses have an outstanding capacity to adapt to new hosts and environments. However, factors that confer adaptability are poorly understood. Viral RNA-dependent RNA polymerases introduce nucleotide substitutions that generate genetic diversity. We hypothesized that selection imposed by hosts and vectors creates a footprint in areas of the genome involved in host adaptation. Here, we profiled genomic and polyprotein variation in all species in the genus *Potyvirus*. Results showed that the potyviral genome is under strong negative selection. Accordingly, the genome and polyprotein sequence are remarkably stable. However, nucleotide and amino acid substitutions across the potyviral genome are not randomly distributed and are not determined by codon usage. Instead, substitutions preferentially accumulate in hypervariable areas at homologous locations across potyviruses. At a frequency that is higher than that of the rest of the genome, hypervariable areas accumulate non-synonymous nucleotide substitutions and sites under positive selection. Our results show, for the first time, that there is correlation between host range and the frequency of sites under positive selection. Hypervariable areas map to the N terminal part of protein P1, N and C terminal parts of helper component proteinase (HC-Pro), the C terminal part of protein P3, VPg, the C terminal part of NIb (RNA-dependent RNA polymerase), and the N terminal part of the coat protein (CP). Additionally, a hypervariable area at the NIb-CP junction showed that there is variability in the sequence of the NIa protease cleavage sites. Structural alignment showed that the hypervariable area in the CP maps to the N terminal flexible loop and includes the motif required for aphid transmission. Collectively, results described here show that potyviruses contain fixed hypervariable areas in key parts of the genome which provide mutational robustness and are potentially involved in host adaptation.

## Introduction

In viruses, host adaptation is an evolutionary process dependent on genetic variation and selection (Obenauer et al., 2006; Bedhomme et al., 2012). Viral RNA-dependent RNA polymerases responsible for viral RNA replication have no proofreading activity and often switch templates. Accordingly, new variants are constantly created by introducing nucleotide substitution (mutations) and recombination (Steinhauer et al., 1992; Garcia-Arenal et al., 2001). Purifying selection reduces the abundance of non-synonymous substitutions causing deleterious effects and favors fixation of those providing adaptive advantages. In contrast, synonymous substitutions are more likely to be maintained (Garcia-Arenal et al., 2003; Wang et al., 2006). The ratio of non-synonymous to synonymous substitutions has been used to determine virus evolution. In several plant and animal viruses, factors that determine virulence, host adaptation, and suppression of defense responses are under positive selection (Moury et al., 2002; Obenauer et al., 2006; Bedhomme et al., 2012).

In the context of genetically diverse hosts and vectors, viruses face selection pressure to maintain functionality and identity of their nucleic acids and proteins in order to interact with pro-viral factors and to evade or suppress antiviral defense (Roossinck, 2003; Longdon et al., 2014). Host and vector genetic diversity and various environmental factors impose heterogeneous selective constraints (Garcia-Arenal et al., 2001; Longdon et al., 2014; Huang et al., 2015). Each combination of host and virus is different and, by favoring different variants, selection contributes to new host adaptation, new strain or species emergence, and host range expansion (Garcia-Arenal et al., 2001; Roossinck, 2003; Huang et al., 2015). Under this model, variation in viral genomes is determined by external and internal constraints imposed by the host, vectors, environmental conditions, and their interactions (Garcia-Arenal et al., 2003; Bedhomme et al., 2012).

Potyviruses (family *Potyviridae*, genus *Potyvirus*) are transmitted by aphids in a non-persistent manner. They form flexuous filamentous particles (700 to 750 nm long) that contain a single copy of positive sense single strand RNA. Genomic RNA is translated into two polyproteins that require proteolytic processing to produce ten mature and one fusion protein essential for replication and movement: P1 (translation, modulator of replication), helper component proteinase HC-Pro (silencing suppression and aphid transmission), P3 (virus replication and movement), P3N-PIPO (cell-to-cell movement), 6K1 (formation of replication vesicles), cytoplasmic inclusion protein (CI, helicase involved in virus movement and replication), 6K2 (formation of replication vesicles), genome-linked protein VPg (translation, virus movement, and replication), NIa-Pro (polyprotein processing), NIb (RNA-dependent RNA polymerase), and CP (virus movement, virion formation and aphid transmission) (Revers and Garcia, 2015; White, 2015; Cui and Wang, 2016).

In potyviruses, genome organization and protein functions are highly conserved (Gibbs and Ohshima, 2010; Revers and Garcia, 2015). However, variable regions have been identified in some species (Johansen et al., 1996; Adams et al., 2005b). In plum pox virus (PPV), the N terminal part of P1 is hypervariable and modulates virus replication, host defense responses, and determines pathogenicity in a host-dependent manner (Maliogka et al., 2012; Pasin et al., 2014). In other potyviruses, variation contributes to host adaptation, host-dependent pathogenicity, vector transmissibility, and viral accumulation in different hosts (Johansen et al., 1996; Tan et al., 2005; Moury and Simon, 2011). Thus, understanding potyvirus variation may provide novel insights into the mechanisms that regulate host adaptation.

Movement of plant material for agricultural purposes contributed to the spread and speciation of potyviruses after an initial radiation event that occurred 6,600 years ago (Gibbs et al., 2008). To date, the genus *Potyvirus* consists of 167 species and has an extensive host range that includes domesticated and wild plants and both monocots and dicots (Wylie et al., 2017). Host range, the number of species that can be infected by a virus, is a reflection of virus adaptability (Rodamilans et al., 2018). The wide host range and word-wide distribution of potyviruses suggest that they have factors that mediate host adaptation. However, factors that confer adaptability to potyviruses are poorly understood. We hypothesized that selection creates a variation foot print in the potyviral genome and can be used to identify viral factors that contribute to host adaptation. In this paper, we profiled variation in potyviruses using single nucleotide polymorphisms (SNPs), nucleotide diversity, and selection analysis. In a complementary approach, we use single amino acid polymorphisms (SAPs) to profile polyprotein variation. Comparison across species showed that the potyviral genome contains hypervariable areas at fixed homologous locations. Hypervariable areas preferentially accumulate nucleotide substitutions, amino acid substitutions, sites under positive selection, and may be determinants of host adaptation.

## Materials and Methods

Computation work was performed on high-performance computing nodes at the University of Nebraska-Lincoln Holland Computing Center (https://hcc.unl.edu/). *In-house* scripts developed for this study are available upon request.

### Genomic and Polyprotein Sequences

Complete genome or polyprotein sequences for all potyviral species represented in GenBank (http://www.ncbi.nlm.nih.gov/) were downloaded on June 28, 2018 using customized scripts based on Entrez Programming Utilities (E-utilities; https://www.ncbi.nlm.nih.gov/books/NBK25500/). For each species, an accession describing the complete genome, and coordinates for each cistron, was used as reference (**Supplementary Table S1**) (**Supplementary Figure S1**). Accessions containing less than 95% of the reference genome or polyprotein length were discarded. To make meaningful statistical comparisons (Shen et al., 2010), only species with at least three accessions were included (81 for RNA and 82 for protein). Fusion protein P3N-PIPO (partially overlaps the P3 open reading frame) was not included in the analyses. *In-house* bioperl and perl scripts were developed to generate a consensus sequence for each species and to determine purine (A and G) and pyrimidine (C and T) content.

### Removal of Recombinant Sequences

RDP4 (http://web.cbio.uct.ac.za/∼darren/rdp.html) (Martin et al., 2015) was used to determine the presence of recombinant nucleotide sequences. Within RDP4, six different methods were used to assess the sequences having recombination breakpoints: RDP, GENECONV, 3Seq, SiScan, MaxChi and BootScan. Default RDP4 settings were used throughout and sequences only with the breakpoints having Bonferroni-corrected p-value ≤ 0.05 were considered as true recombinants and removed subsequently. Accessions containing recombinant sequences were removed and were not part of the analyses.

### Potyvirus Phylogeny

A tree-based progressive method was used in MAFFT version 7.3 (Multiple Alignment (https://mafft.cbrc.jp/alignment/software/) to generate Multiple Sequence Alignments (MSA) (Abdel Azim et al., 2011; Katoh and Standley, 2013). Gaps were deleted from the alignment using GapStrip/Squeeze v2.1.0 (http://www.hiv.lanl.gov/content/sequence/GAPSTREEZE/gap.html). Based on the lowest Bayesian Information Criterion (BIC) (Lefort et al., 2017), the best-fit nucleotide and protein substitution model was estimated using Smart Model selection in PhyML. Maximum likelihood phylogenetic trees for all potyviruses were estimated in PhyML 3.0. Trees were visualized and customized using Figtree (http://tree.bio.ed.ac.uk/software/figtree/) (Rambaut, 2009).

### Polymorphism Analysis

For each virus species, the genomic or polyprotein sequence alignment (.aln) file obtained from MAFFT was used for identification of SNPs or SAPs with *SNP-sites version 2.4.1* (https://github.com/sanger-pathogens/snp-sites) (Page et al., 2016)*. T*he nature and position of each substitution was extracted in a variant call format (VCF). Using VCFtools (Danecek et al., 2011), SNP and SAP were obtained in a 50-nt or amino acid window and normalized to the length of the window. For each virus, a variation index was calculated by normalizing total SNPs or SAPs to the length of the genome or polyprotein, respectively. For all potyviruses with detectable variation, and for the viruses with the highest number of SNPs and represented by ten or more accessions, a local regression curve was fitted between the number of SNPs or SAPs, and the number of accessions using ggplot2 in R. The geom_smooth function was applied with the method "loess" (Wickham, 2009). In an alternative approach, alignment files in nexus format for all genomic sequences were used to determine pairwise nucleotide diversity (Pi) in a 50-nt sliding window using the Tajima's D test in DnaSP 5.10.1 (Rozas, 2009). To establish a variation threshold in both analyses, a 99% confidence interval was estimated using the Z-score [X ± (Z*s*√n)] (Hazra, 2017). In this equation, X is the mean, Z is the Z value with 99% confidence, s is the standard deviation, and n is the number of sequence accessions.

### Sequence Variation Clusters

SNPs or SAPs were subjected to hierarchical clustering using the ClustVis package in R (Wickham et al., 2013). Groups were generated by first finding the shortest link among all of the data points (species or coordinates) and then combining those points into a virus group as a cluster.

### Genome-Wide Distribution of Substitutions

To visualize variation along the genome or polyprotein, for each virus, all available accessions were aligned and identity plots generated in Geneious version 8.0 (https://www.geneious.com/). For selected polymorphic areas, a sequence logo was obtained from the same alignment. A 99% confidence interval for SNPs, SAPs and Pi was estimated and plotted for each potyvirus species. 

### Selection Analysis

Full-length coding sequences of the 16 most and 16 least variable potyviruses were aligned with MAFFT. Nucleotide ambiguities within the sequences were discarded using a custom bash script. The resulting alignment file was used to obtain the rate of non-synonymous and synonymous changes at each site based on Single-likelihood ancestor counting (SLAC) and MEME using HyPhy (Obenauer et al., 2006). A significance level ≤0.05 and >0.95 posterior probability was used for both SLAC and MEME (Murrell et al., 2012). Only those sites detected by both methods were considered under positive selection. Abundance of positive and negative selection was normalized to the number of codons per cistron.

### Host Range

Using a custom bash script, GenBank files used in the SNP analysis were parsed to get the host range for each viral species. For each accession, the name of the host from which the sequence was generated was extracted and the frequency of each host determined for each virus. A Pearson correlation analysis was performed between the host range of each potyvirus and the positive selection sites.

### GC Content

A bioperl script was used to calculate the GC content using a 50-nt window (Gao and Zhang, 2006).

### Nucleotide and Amino Acid Substitution Profiles

SNPs were classified as transitions or transversions (Zhao et al., 2006). All possible amino acid substitutions were evaluated. A custom bash script was developed to calculate their frequency from the VCF file. The five most abundant SNPs and SAPs types from each virus were used to generate a matrix and color assignment for the top four substitutions from each virus. For each potyvirus consensus polyprotein, an amino acid profile was obtained *via* COPid web-server (http://crdd.osdd.net/raghava/copid/help.html) (Kumar et al., 2008).

### Codon Usage Bias

CodonW 1.4.4 was used to determine Relative Synonymous Codon Usage (RSCU) (Bera et al., 2017) using the consensus sequence for each potyvirus. Termination codons, AUG, and UGG encoding Met and Trp, respectively were removed from dataset because they do not have synonymous codons and do not contribute to codon bias. Codons with a RSCU value of >1.6 were considered over-represented, whereas codons with a RSCU value of <0.6 were considered underrepresented. Codons used at an equivalent level (no bias) have a RSCU value of 1 (Wong et al., 2010).

### Phylogenetic Analysis and Variation Maps

For selected viruses, all full-length genomic and polyprotein sequences available from GenBank were used to generate a phylogram in PhyloXML format with MAFFT. GraPhlAn (http://segatalab.cibio.unitn.it/tools/graphlan/) (Asnicar et al., 2015) was used to create an annotated phylogram containing layers indicating insertions at the NIb-CP junction, country of origin, and host.

### Coat Protein Structure Model

A three-dimensional model of the CP of the five potyviruses with the most variation was generated using Phyre2 (http://www.sbg.bio.ic.ac.uk/phyre2/html/page.cgi?id=index) under intensive mode (Kelley et al., 2015). A custom bash script was used to extract the CP amino acid sequence based on the coordinate information in GenBank records. Models (in.pdb format) for two viruses or isolates were superimposed using Chimera v1.13 (https://www.cgl.ucsf.edu/chimera/). The TM-Score was used for structure alignment measurement.

### DAG Motif Prediction and Variation Within the Potyvirus Coat Protein

A custom bash script was used to calculate the frequency of the Asp-Ala-Gly (DAG) motif in the CP. Positive selection sites were flagged on the N-terminal, core, and C-terminal part of CP using the marker option within the graphic tool available on NCBI. Variations in location of DAG motif or in amino acid sequence was determined by alignment with Geneious version 8.0.

## Results

### Genomic and Polyprotein Sequences

A total of 15,668 genomic RNA and 16,397 polyprotein non-recombinant sequences for 95 potyviruses were obtained from NCBI. A total of 2,198 full-length RNA and 2,200 polyprotein accessions were included in the analyses. Potyviral genomic RNA varied from 9,300 to 10,800 nt in length, with an average genomic and polyprotein length of 9,799 nt and 3,125 amino acids, respectively (**Supplementary Table S1**). The Chargaff's purine–pyrimidine equilibrium (Aryal et al., 2012) was not detected. Instead, the potyvirus genome is biased towards purines. The average purine (G + A, 55.42%) to pyrimidine (U + C, 44.57%) ratio (1.24) was significantly higher than 1.0 (p-value ≤ 0.00001) (**Supplementary Table S2**).

### Potyvirus Phylogeny

Viruses depend on host factors at all parts of the infection cycle (Garcia-Ruiz, 2018). However, they must suppress or evade antiviral immunity initiated by host factors to establish infection (Csorba et al., 2015). These interactions result in virus and host co-evolution. Additionally, mutations in viral genomes are associated with host specificity and with host shifts (Bedhomme et al., 2012; Longdon et al., 2014). This model suggest that closely related viral species would infect closely related host plants. To test this hypothesis, a nucleotide- and a polyprotein-based phylogeny were obtained. Families *Solanaceae*, *Poaceae*, *Fabaceae*, and *Cucurbitaceae* were the most frequent (**Supplementary Figure S2**). Both the nucleotide- and the polyprotein-based approaches grouped viruses into similar clusters that were associated with the botanical family of their hosts. This is consistent with the model that potyviruses are co-evolving with and adapting to their hosts. Thus, we hypothesized that, during host adaptation, selection imposed by the host leaves a foot print in the potyviral genome. This model predicts that potyviruses contain areas of the genome that determine host adaptation.

### Nucleotide and Polyprotein Variation

**Figure 1:**
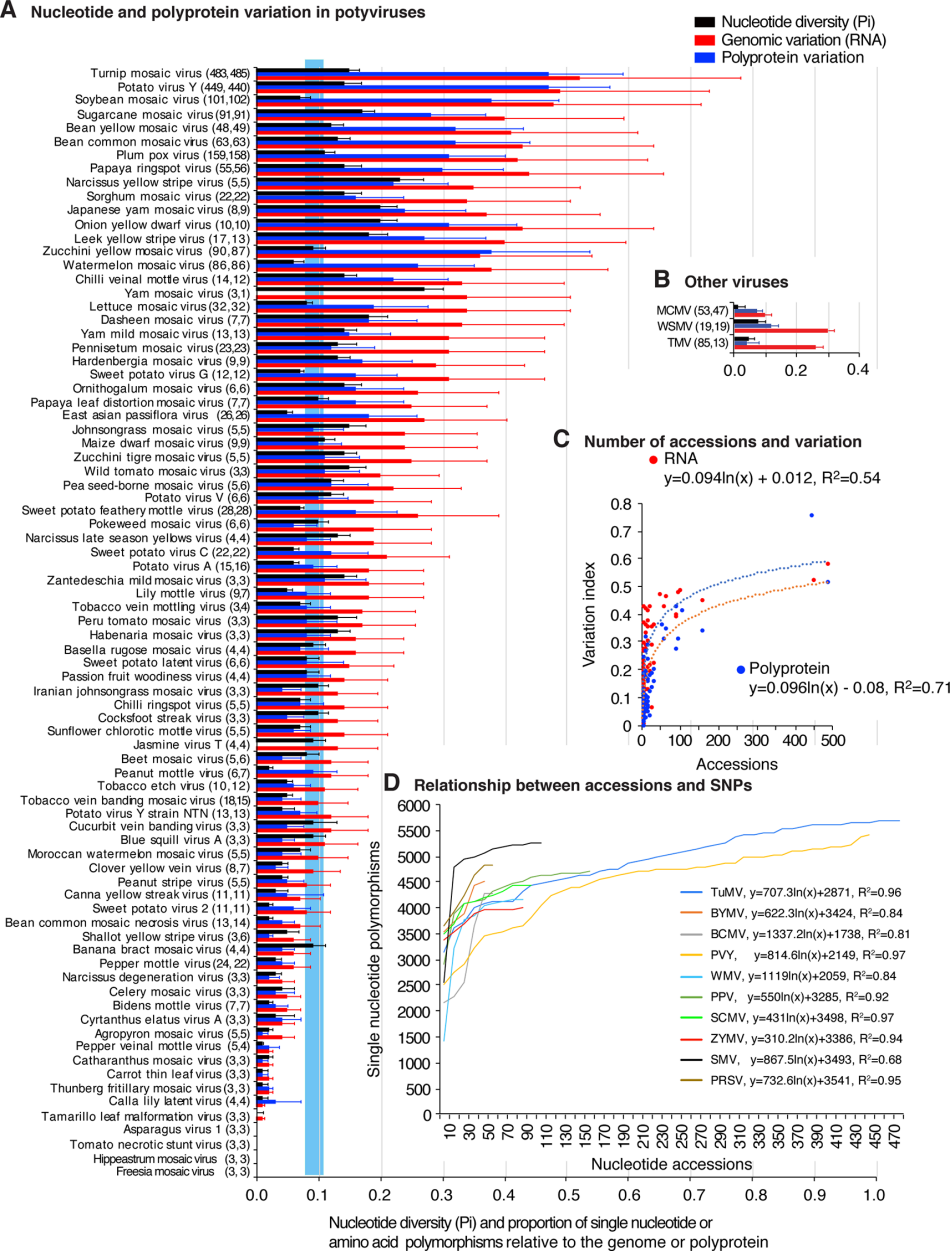
Single nucleotide and amino acid polymorphism in potyviruses. Respect to the genome or polyprotein, bars represent the proportion of polymorphic sites (number of single nucleotide or amino acid polymorphism/length of the genome or polyprotein). For each species, the number of nucleotide and polyprotein accessions are indicated in parenthesis. Species with less than three accessions were not included. **(A)** Nucleotide and polyprotein variation in potyviruses, as determined by nucleotide diversity (Pi), genomic variation index and polyprotein variation index. Bars represent the average and standard error for each species and were estimated for the entire genome on a 50-nt or 50-amino acid interval. The blue vertical line represents the mean Pi and a 99% confidence interval for all potyviruses with detectable nucleotide variation. **(B)** Nucleotide and protein variation is three non-potyviruses used for comparison, maize chlorotic mottle virus (MCMV) genome and p111, wheat streak mosaic virus (WSMV) genome and polyprotein, and tobacco mosaic virus (TMV) genome and replicase protein. **(C)** Relationship between the number of sequence accessions available and nucleotide or polyprotein sequence variation. Variation and number of accession are as indicted in panel **(A)**. **(D)** Relationship between the number of sequence accessions and number of SNPs for the top 10 viruses with the most variation. Accessions available were processed in increments of 10. Parameters of a regression line are indicated for each virus.

To measure and map nucleotide variation, genome-wide SNPs and nucleotide diversity (Pi) analyses were used. SAPs were used to measure and map polyprotein variation. Pi measures nucleotide substitutions and corrects for the number of accessions (Rozas, 2009). Results show that 61 of the 81 potyviruses exhibit higher Pi (**Figure 1A**) than the genetically stable viruses used for comparison: tobacco mosaic virus (TMV), wheat streak mosaic virus (WSMV) and maize chlorotic mottle virus (**Figure 1B**). SNPs and SAPs were detected in 79 and 76 potyviruses, respectively (**Figure 1** and **Supplementary Table S3**). For turnip mosaic virus (TuMV) and potato virus Y (PVY), nucleotide variation was 0.52 and 0.49, which means that approximately 50% of the nt positions in the genome are polymorphic.

Polyprotein variation was expressed using a polyprotein variation index obtained in a similar way. Results show that 48 of the 81 potyviruses exhibit higher polyprotein variation (**Figure 1A**) than genetically stable viruses used for comparison (**Figure 1B**). Amino acid and nucleotide variation follow a similar pattern (**Figure 1A**).

Our potyviral species dataset is represented by viruses with 3 to 485 sequence accessions (**Figure 1A**). If these sequences originated from a random sample, it would be logical to expect that a higher number of sequences would increase the chances of finding polymorphic nucleotide or amino acid sites or new hosts. It could also be expected that the difference in accession number could potentially impact diversity estimates, positive selection sites, and host range. However, the relationship between abundance of nucleotide or amino acid polymorphisms and the number of sequences available follows a rarefaction curve (Chiarucci et al., 2009) modeled by a logarithmic function in which the number of polymorphisms reaches a point of saturation at approximately 150 accessions (**Figure 1C** and **Supplementary Figure S3A**). For the viruses with the most SNPs and represented by 10 or more accessions, SNPs were determined by increments of 10 accessions, without replacement. Results show that, for individual species, as for the entire group of potyviruses with detectable variation, the relationship between abundance of nucleotide polymorphisms and the number of sequences available follows a rarefaction curve modeled by a logarithmic function. Most of the potyviruses are represented by less than 150 accessions (**Figure 1A**) and the point of saturation for the number of polymorphisms is different for each virus (**Figure 1D** and **Supplementary Figure S3B**). Thus, instead of random sub-sampling, we analyzed all sequences available for each potyvirus species and estimated genomic variation using Pi to normalize for the number of accessions (Rozas, 2009).

### Hypervariable Areas in the Potyviral Genome

Nucleotide substitution may accumulate randomly or be concentrated in particular areas of the genome. To distinguish the difference, we performed a two-way hierarchical cluster analysis of SNPs. Viral species were clustered into low (58 species) and abundant (21 species) nucleotide variation groups. The 5' UTR, the N terminal part of P1, and the N and C terminal parts of HC-Pro formed a cluster with the highest variation. Other areas with high variation included the C terminal part of P3, the NIb-CP junction, and VPg (**Supplementary Figure S4**).

**Figure 2:**
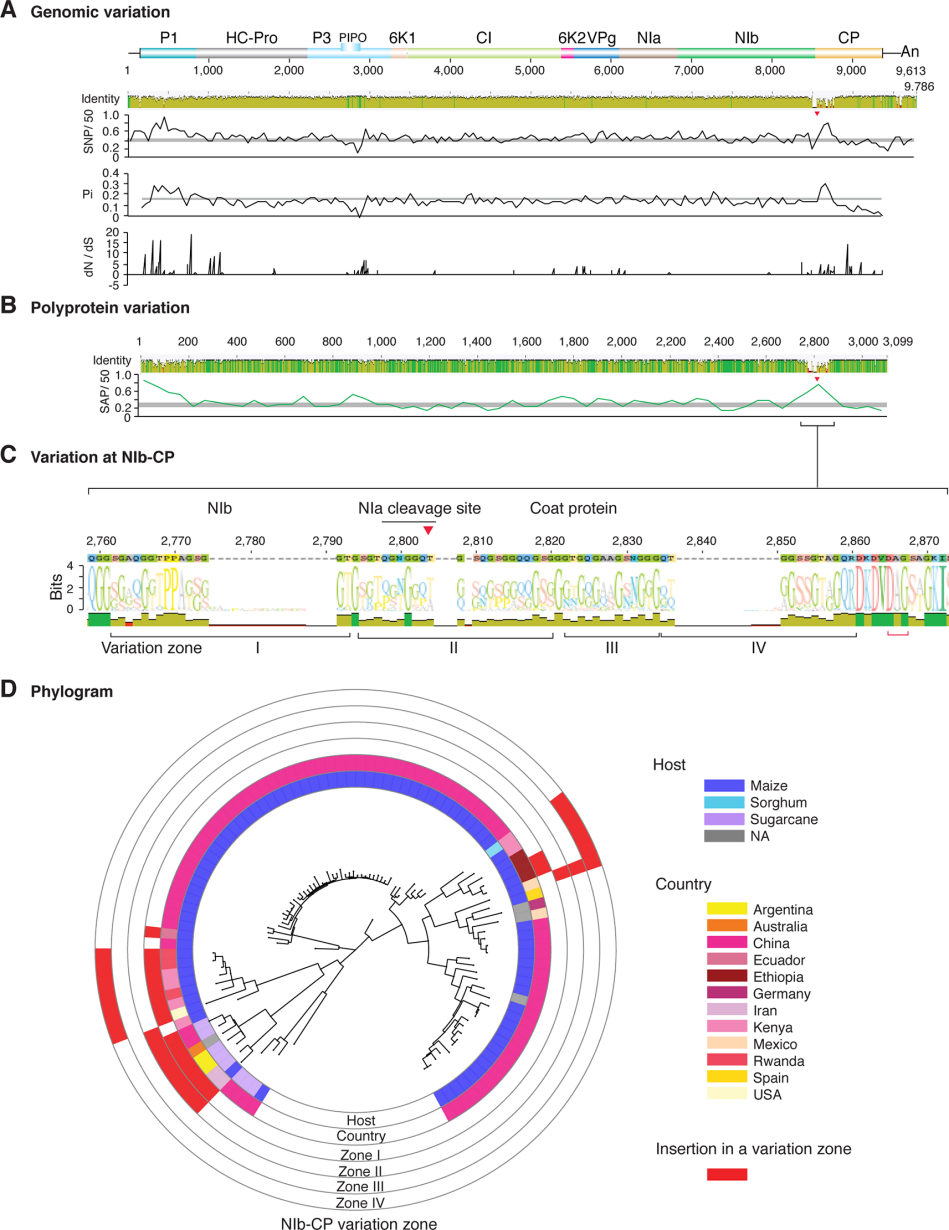
Nucleotide and polyprotein variation in sugarcane mosaic virus. Accessions available were aligned to generate an identity plot. Coordinates are based on accession JX188385.1. **(A)** Genome-wide nucleotide variation. Single nucleotide polymorphisms (SNP) were estimated and normalized in a 50-nt window. The 5' and 3' UTR were included. Nucleotide diversity (Pi) and dN/dS ratio were estimated for the open reading frame in a 50-nt window or at each codon, respectively. The average and a 99% confidence interval are represented by a horizontal gray line. Arrow heads point to the NIb-CP junction. **(B)** Polyprotein variation. Single amino acid polymorphisms (SAP) in the polyprotein were estimated and normalized in a 50-amino acid window. The average and a 99% confidence interval are represented by a horizontal gray line. **(C)** Variation at the NIb-CP junction. The sequence logo includes the consensus. A red arrowhead points to the NIa-Pro cleavage site. The DAG motif is marked with a red bracket. Variation zones are numbered. **(D)** Phylogram. The phylogenetic tree in the center was generated using GraPhlAn using the full-length polyprotein sequences. Rings indicate the host, country of origin, and variation zones. A red mark indicates insertion at a variation zone.

**Figure 3:**
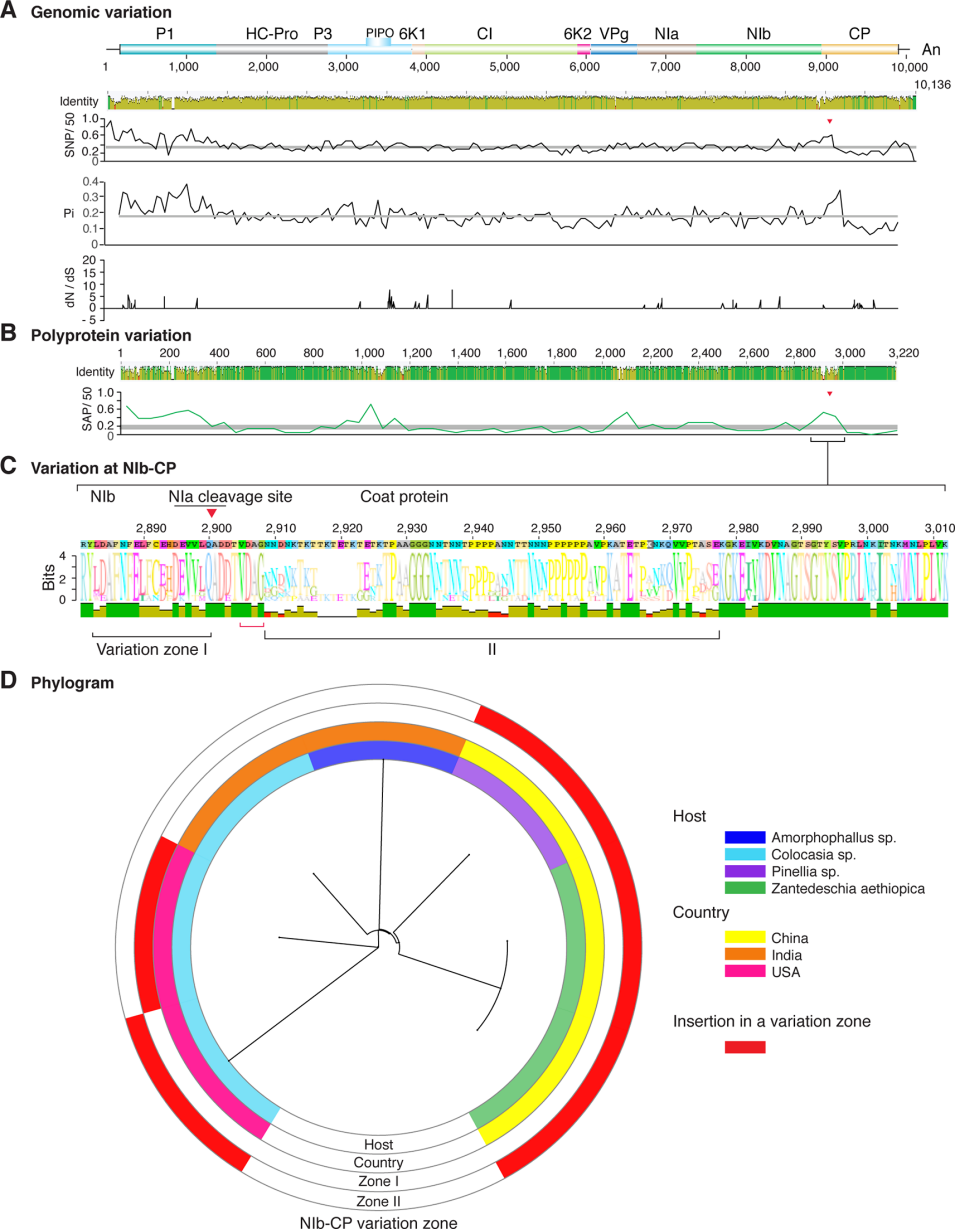
Nucleotide and polyprotein variation in dasheen mosaic virus. Genome organization is shown to scale using accession JX083210 as reference. Labels are as in **Figure 2**.

**Figure 4:**
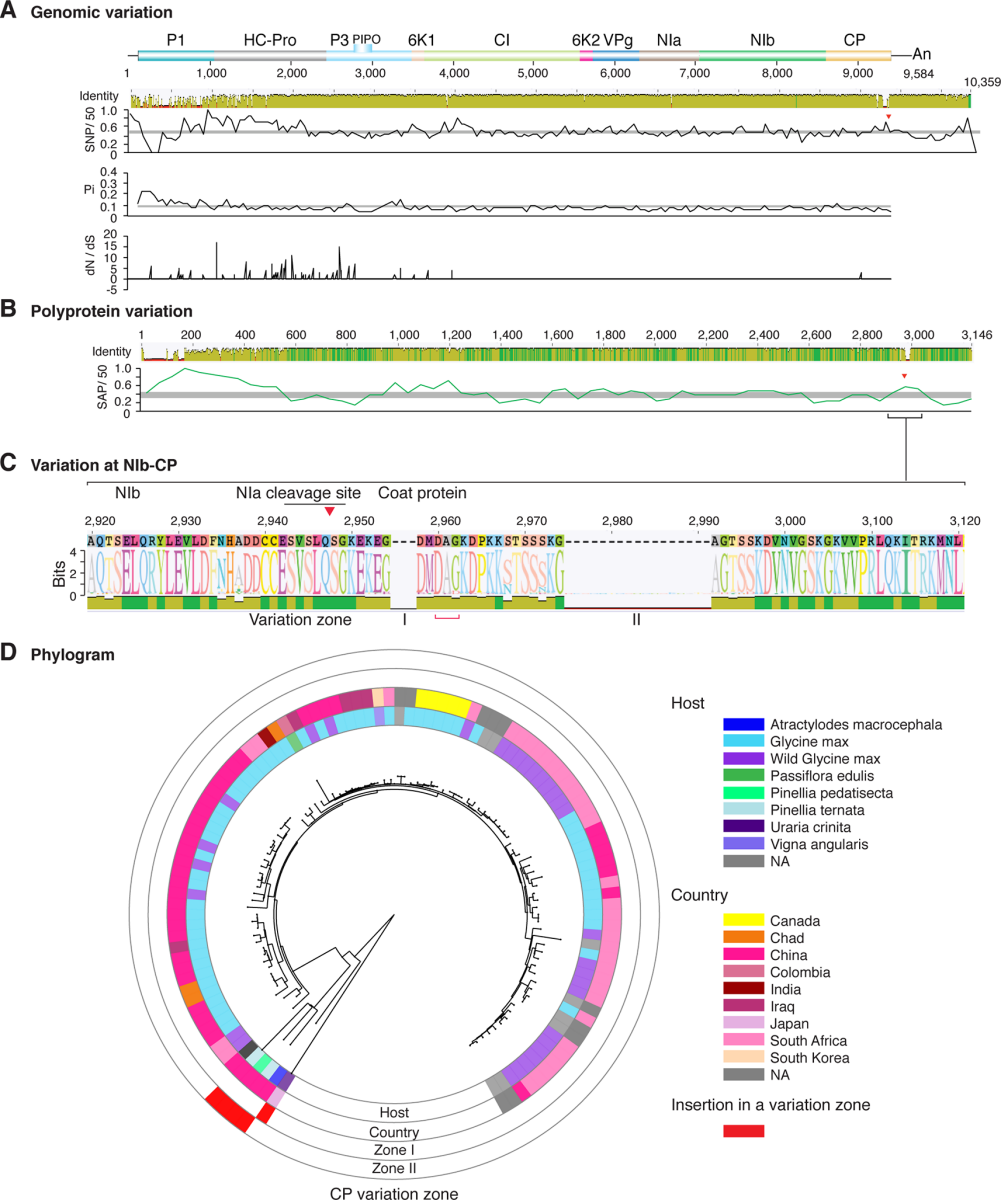
Nucleotide and polyprotein variation in soybean mosaic virus. Genome organization is shown to scale using accession KY986929 as reference. Labels are as in **Figure 2**.

**Figure 5:**
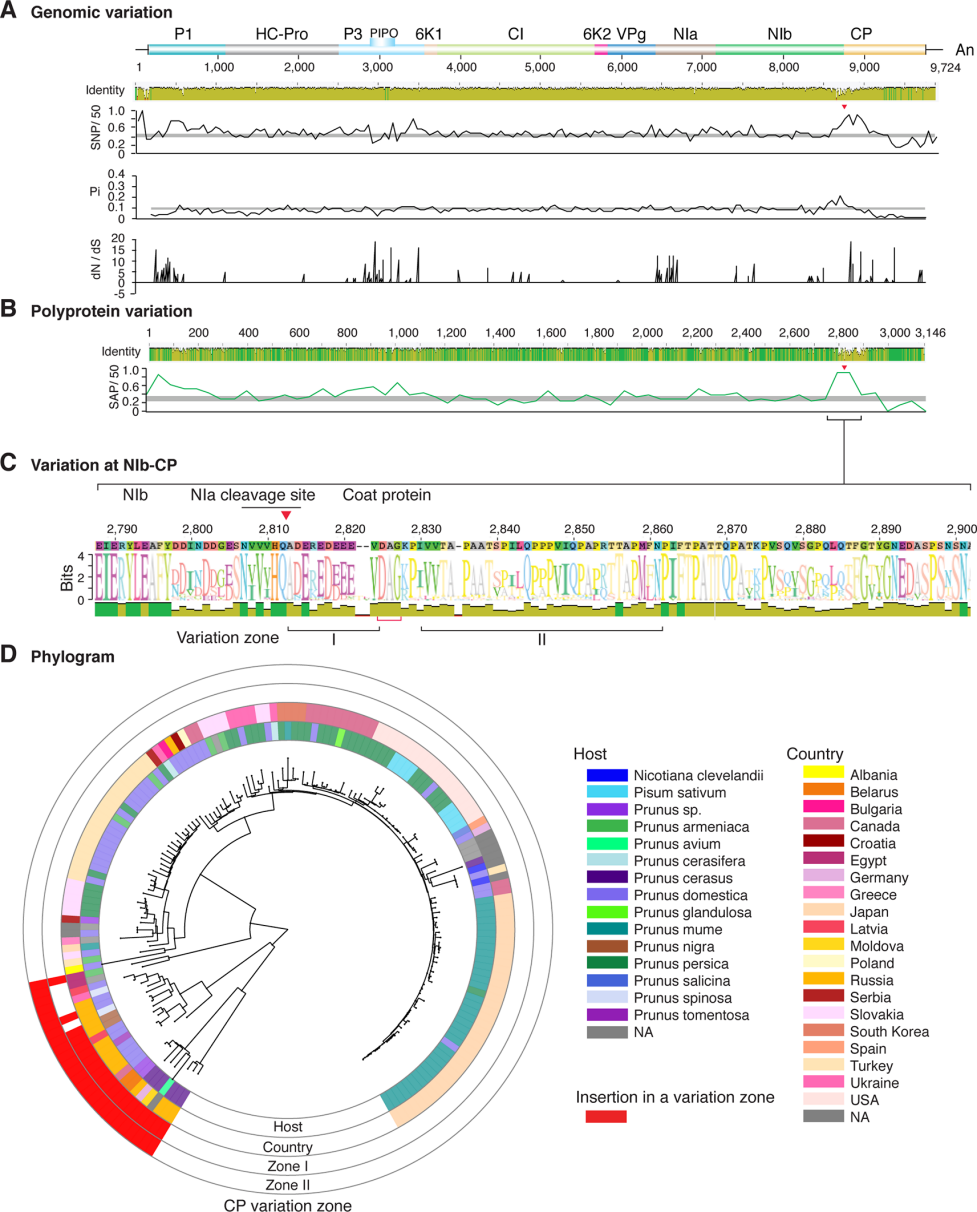
Nucleotide and polyprotein variation in plum pox virus. Genome organization is shown to scale using accession MF370984.1 as reference. Labels are as in **Figure 2**.

**Figure 6:**
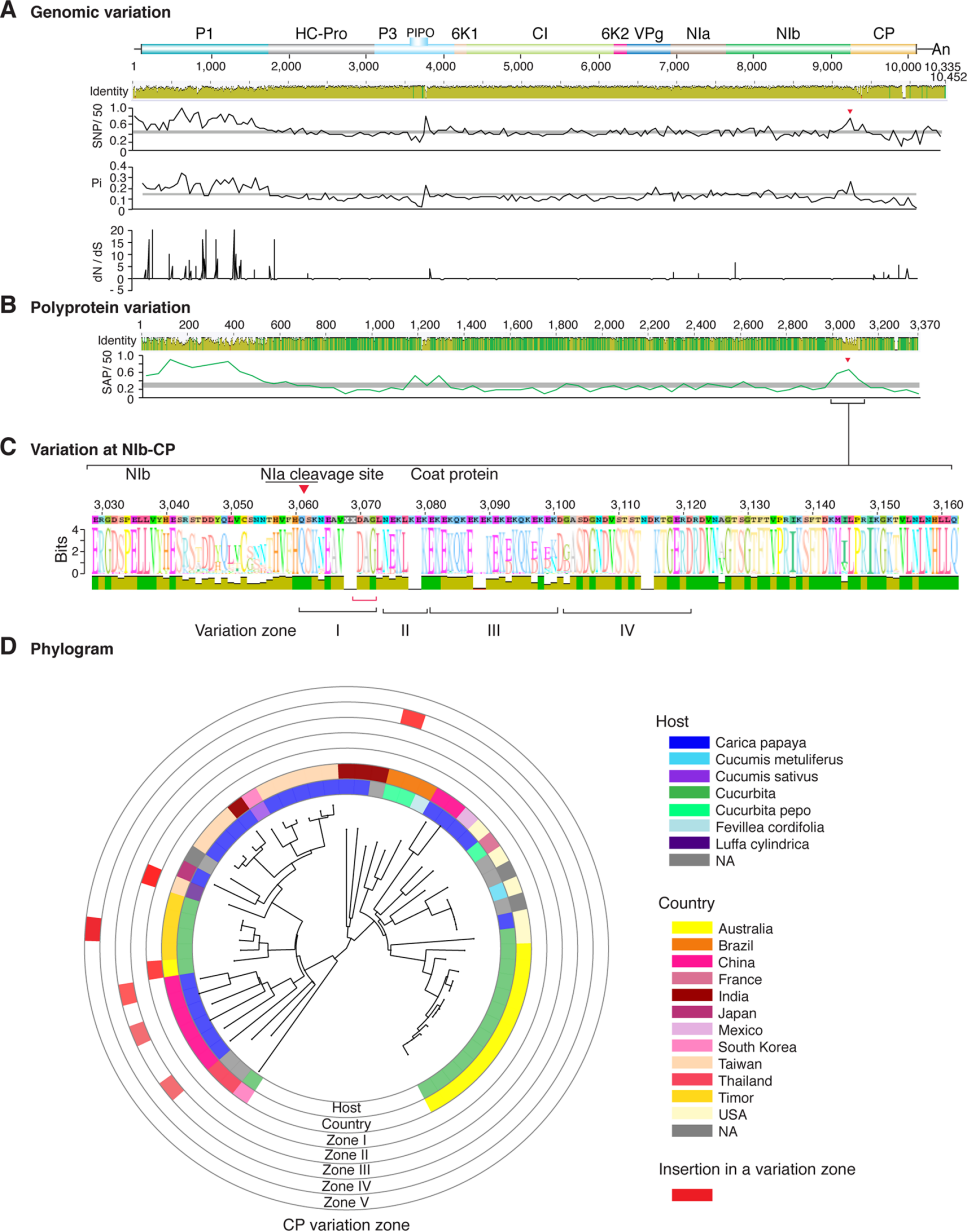
Nucleotide and polyprotein variation in papaya ringspot virus. Genome organization is shown to scale using accession KP462721.1 as reference. Labels are as in **Figure 2**.

**Figure 7:**
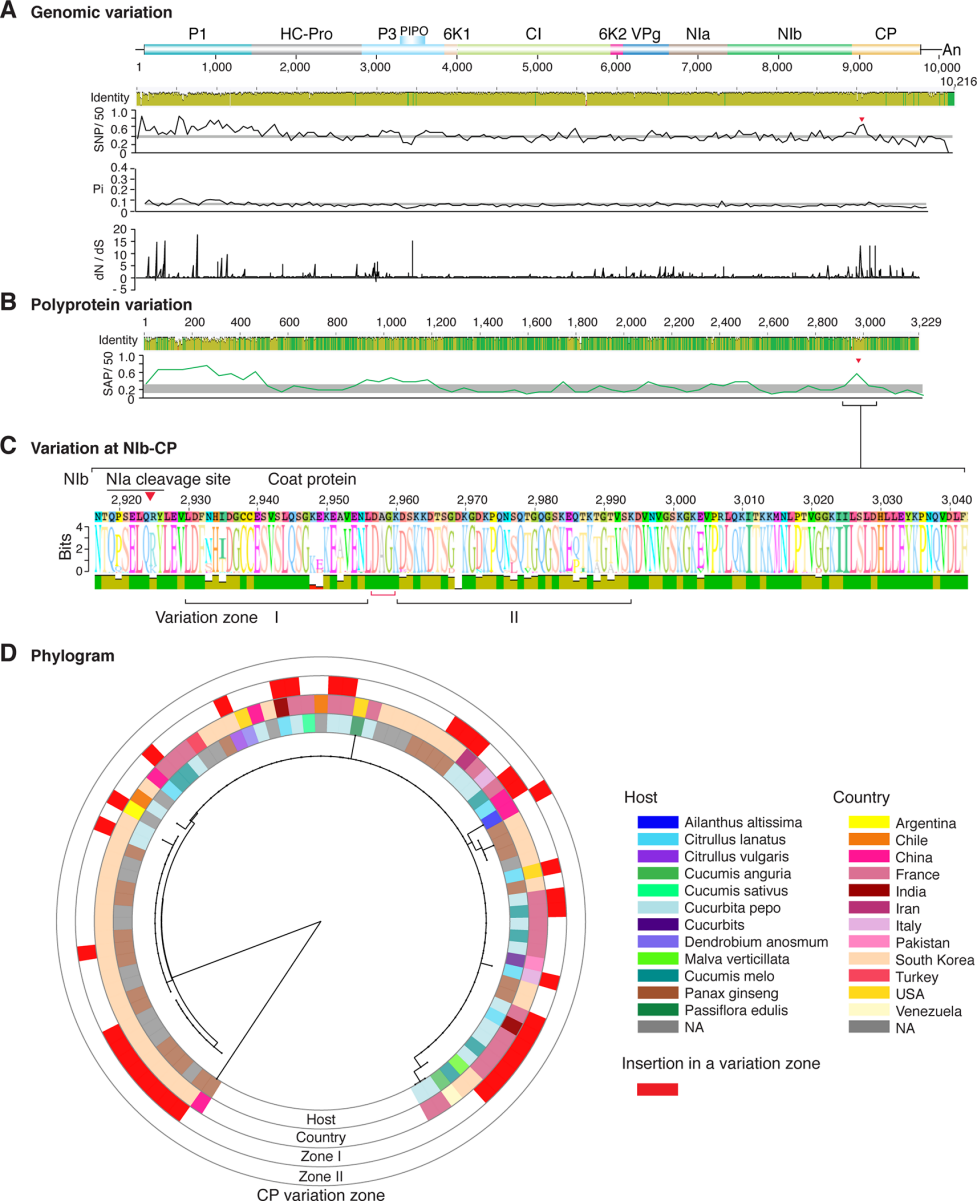
Nucleotide and polyprotein variation in watermelon mosaic virus. Genome organization is shown to scale using accession KX926428.1 as reference. Labels are as in **Figure 2**.

**Figure 8:**
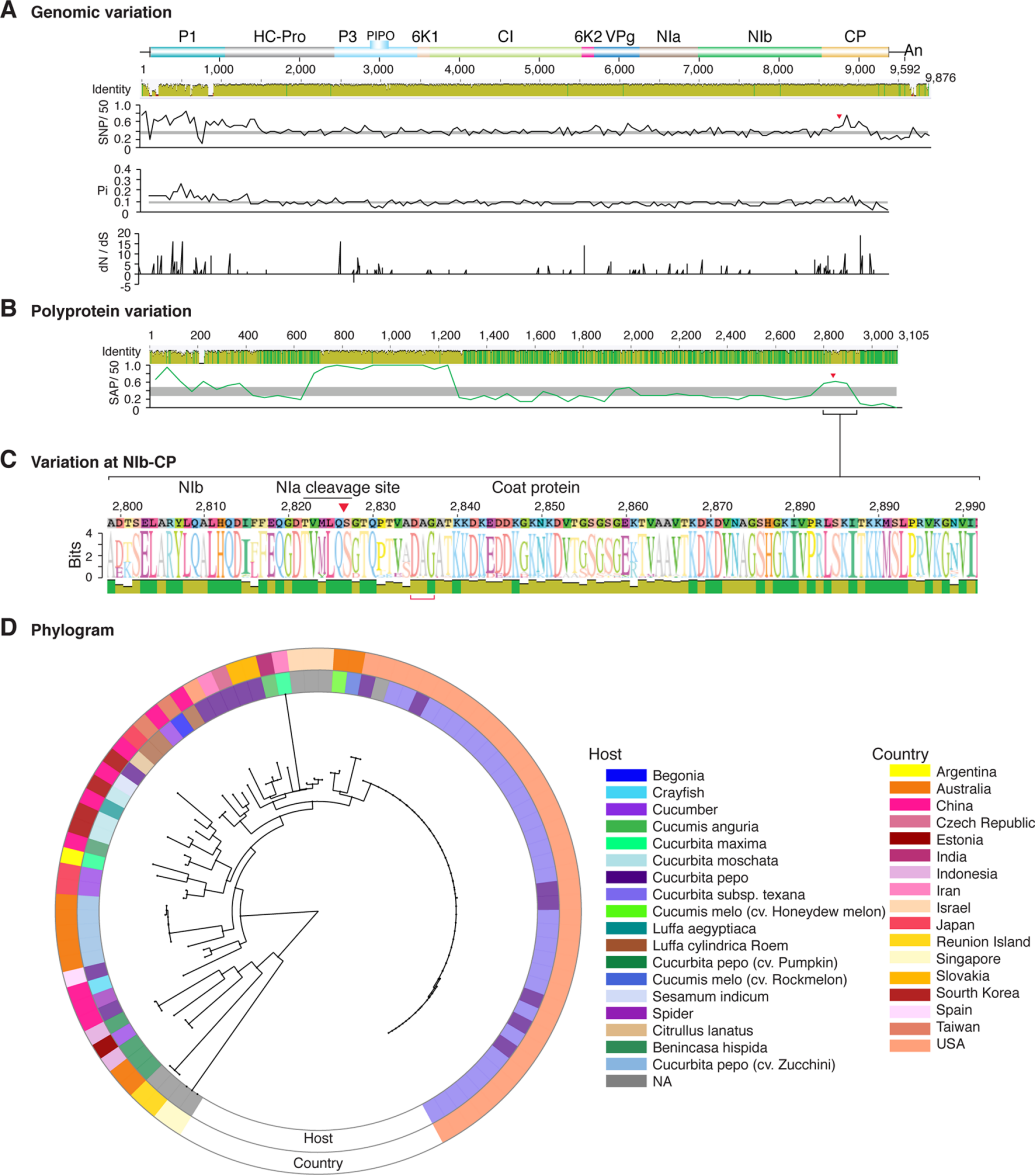
Nucleotide and polyprotein variation in zucchini yellow mosaic virus. Genome organization is shown to scale using accession KX499498.1 as reference. Labels are as in **Figure 2**.

**Figure 9:**
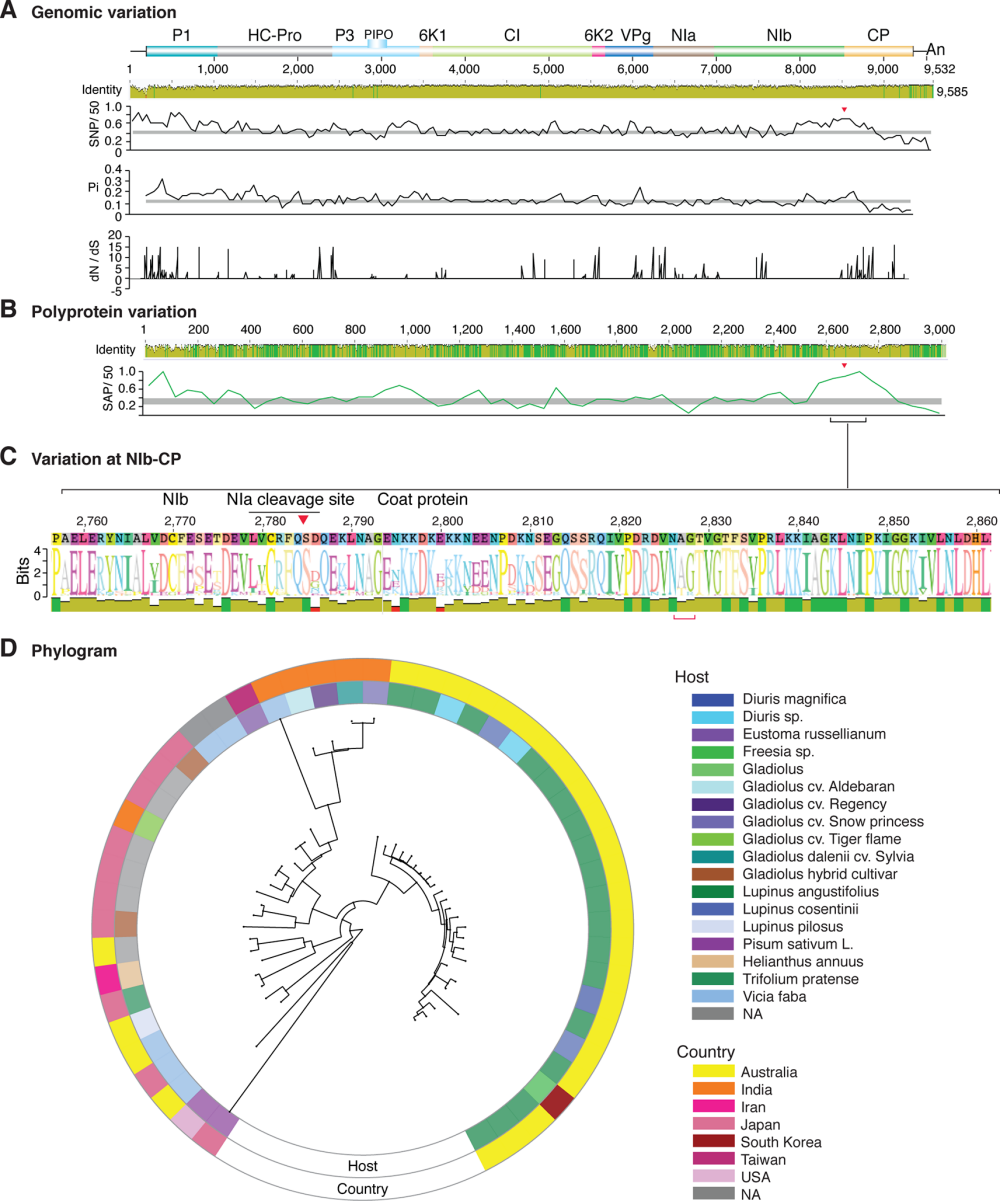
Nucleotide and polyprotein variation in bean yellow mosaic virus. Genome organization is shown to scale using accession NC_003492.1 as reference. Labels are as in **Figure 2**.

**Figure 10:**
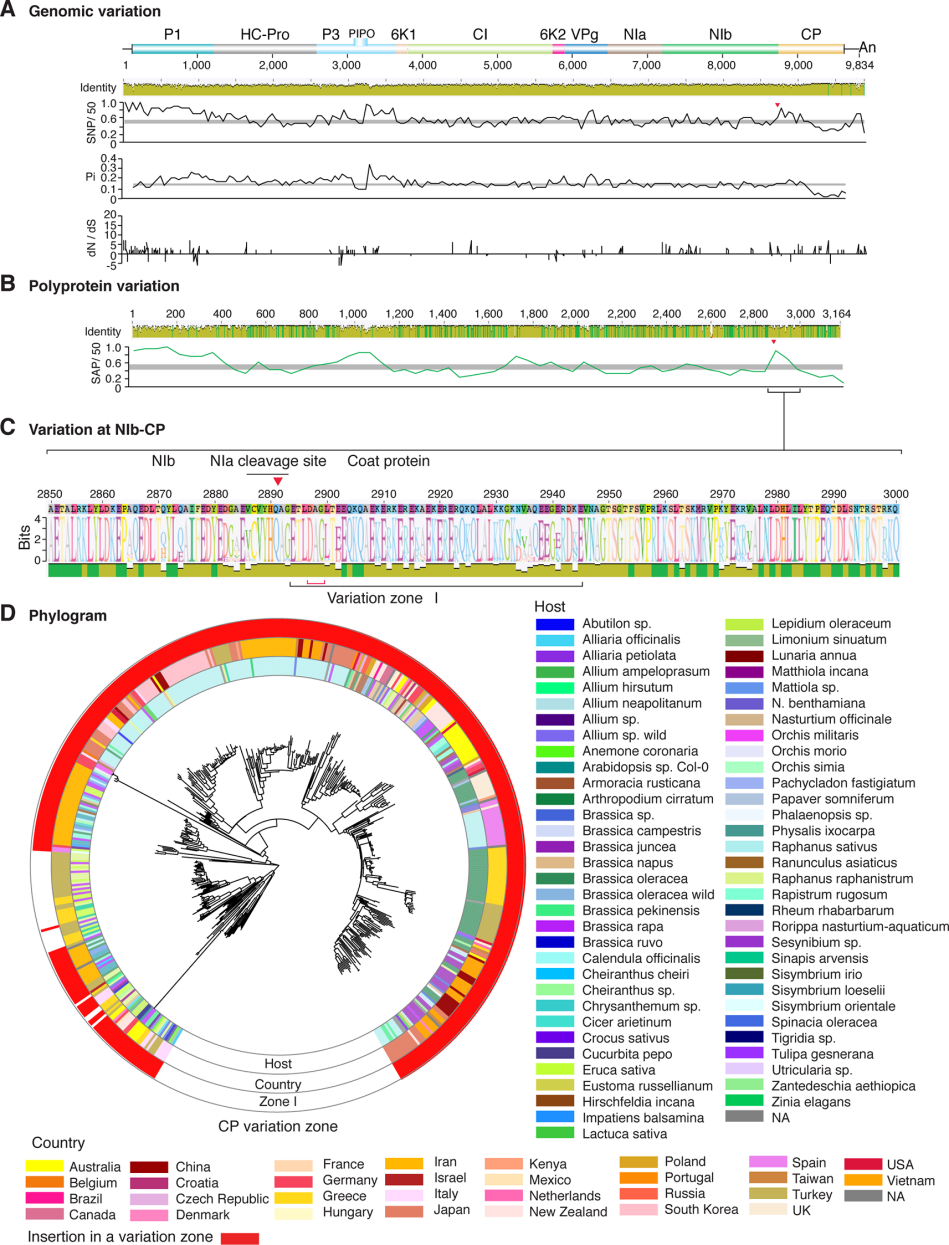
Nucleotide and polyprotein variation in turnip mosaic virus. Genome organization is shown to scale using accession AF169561.2 as reference. Labels are as in **Figure 2**.

**Figure 11:**
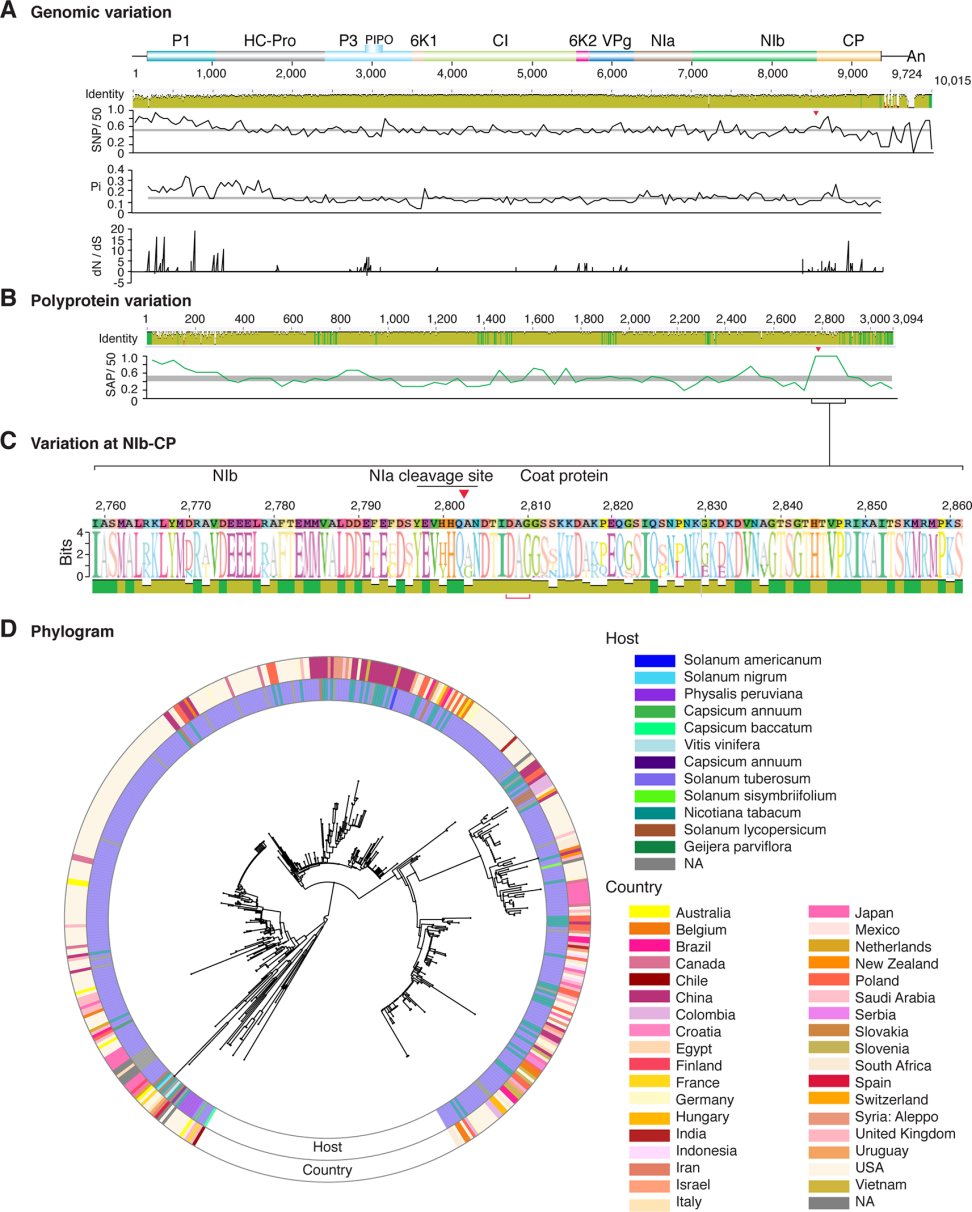
Nucleotide and polyprotein variation in potato virus Y. Genome organization is shown to scale using accession MG591487.1 as reference. Labels are as in **Figure 2**.

To visualize the distribution of nucleotide substitutions, a genome-wide map was obtained for each virus. SNPs and Pi obtained were plotted with respect to the virus genome. In a complementary approach, individual sequences were aligned to generate an identity plot (**Figures 2**–**11**). Genome-wide variation maps were generated for the 16 potyviruses with the highest genomic variation index (**Supplementary Figure S5**). Results identified areas containing nucleotide substitutions, insertions, or deletions across the genome. Comparison across potyviruses showed that, similar to the two-way clustering, nucleotide substitutions preferentially accumulate at the 5'UTR, the N terminal part of P1, N and C terminal parts of HC-Pro, the C terminal part of P3, VPg, C terminal part of NIb, and the N terminal part of the CP (**Figures 2**–**11** and **Supplementary Figure S5**). Less variation was observed in the area overlapping with P3N-PIPO, a highly-conserved protein essential for potyvirus movement (Vijayapalani et al., 2012). These results show that the distribution of nucleotide substitutions in the potyviral genome is not random.

### Polyprotein Hypervariable Areas

We performed a two-way hierarchical cluster analysis (**Supplementary Figure S6**), and polyprotein variation maps were generated to visualize the distribution of amino acid substitutions. P3N-PIPO was not part of the analysis because it is a fusion protein that overlaps P3, and P3N-PIPO specific coordinates were not provided for most of the accessions. Comparison across species showed that amino acid substitutions mainly occurred at the N terminal part of P1, the N and C terminal parts of HC-Pro, the C terminal part of P3, VPg, the C terminal part of NIb and the N terminal part of the CP (**Figures 2**–**11** and **Supplementary Figure S7**). These areas were also detected by the nucleotide substitution and nucleotide diversity analyses described above.

Amino acid substitutions in protein CI and NIa-Pro followed contrasting patterns across potyviruses. Some species, such as sugarcane mosaic virus (SCMV), showed low variation in CI (**Figure 2**), whereas TuMV (**Figure 10**), PVY (**Figure 11**), and chilli veinal mottle virus (CVMV), **Supplementary Figure S7**) harbor high variation at the C terminal part of CI. In contrast, soybean mosaic virus (SMV, **Figure 4**) and ZYMV (**Figure 8**) harbor variation at the N terminal part of CI.

**Figure 12:**
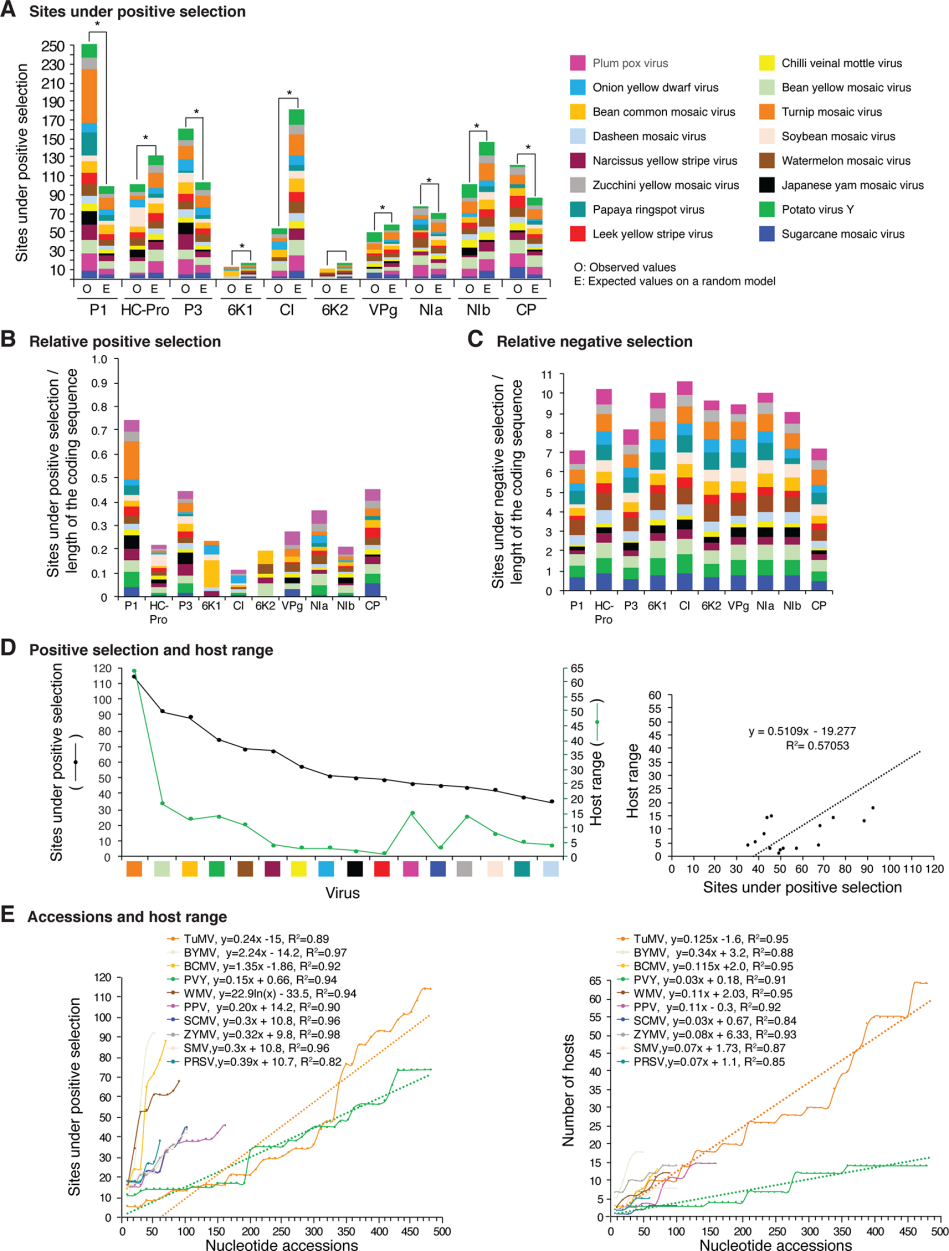
Frequency of sites under positive or negative selection per cistron and their relation with host range in the top 16 viruses with the most variation. Only positive selection sites identified with both SLAC and MEME were counted. Sites under negative selection were detected with SLAC. Virus species are color coded. **(A)** Number of sites under positive selection compared to the expected randomly (sites per cistron/total for the open reading frame, and normalized to the length of the cistron). * indicates significant differences with p-value ≤0.001 as calculated by the Chi-square test. **(B)** Per virus and per cistron, sites under positive selection normalized to the number of codons. **(C)** Sites under negative selection per cistron and per virus, normalized to the number of codons. **(D)** Relationship between sites under positive selection and host range. **(E)** Relationship between number of accession and number of hosts. The left panel includes all viruses in panel **(D)**. The panel on the left shows number of accessions in increments of 10. For each species, parameters of a regression line are indicated.

NIa-Pro shows higher than the average genomic variation in SCMV (**Figure 2**), PPV (**Figure 5**), leek yellow stripe virus (LYSV), bean common mosaic virus (BCMV), and onion yellow dwarf virus (OYDV) (**Supplementary Figure S7**). For several viruses, NIa-Pro accumulates sites under positive selection at a frequency higher than randomly expected (**Figure 12A**). However, variation at NIa-Pro did not map to a particular area. In LYSV and BCMV, variation is higher in the central part of NIa-Pro, whereas in OYDV and PPV, variation is higher at the N terminus.

The genome-wide and polyprotein-wide analyses described above showed that nucleotide and amino acid substitution are not randomly distributed in potyviruses. Independently, nucleotide diversity, nucleotide and amino acid substitution analyses showed that substitutions accumulate at a frequency higher than average (p-value ≤ 0.01) at the N terminal part of P1, the N and C terminal parts of HC-Pro, the C terminal part of P3, VPg, the C terminal part of NIb, and the N terminal part of the CP (**Figures 2** **–** **11** and **Supplementary Figures S5** and **S7**). We refer to these areas as hypervariable.

### Potyviral Proteins Under Positive Selection

In viruses, sites under positive selection provide an evolutionary advantage, support adaptation to new hosts, and contribute to an increased host range (Schneider and Roossinck, 2001; Obenauer et al., 2006; Bedhomme et al., 2012). If hypervariable areas in potyviruses are related to host adaptation, positive selection sites will preferentially accumulate in the same cistrons. We tested this hypothesis using SLAC and MEME (**Supplementary Table S4**).

**Figure 13:**
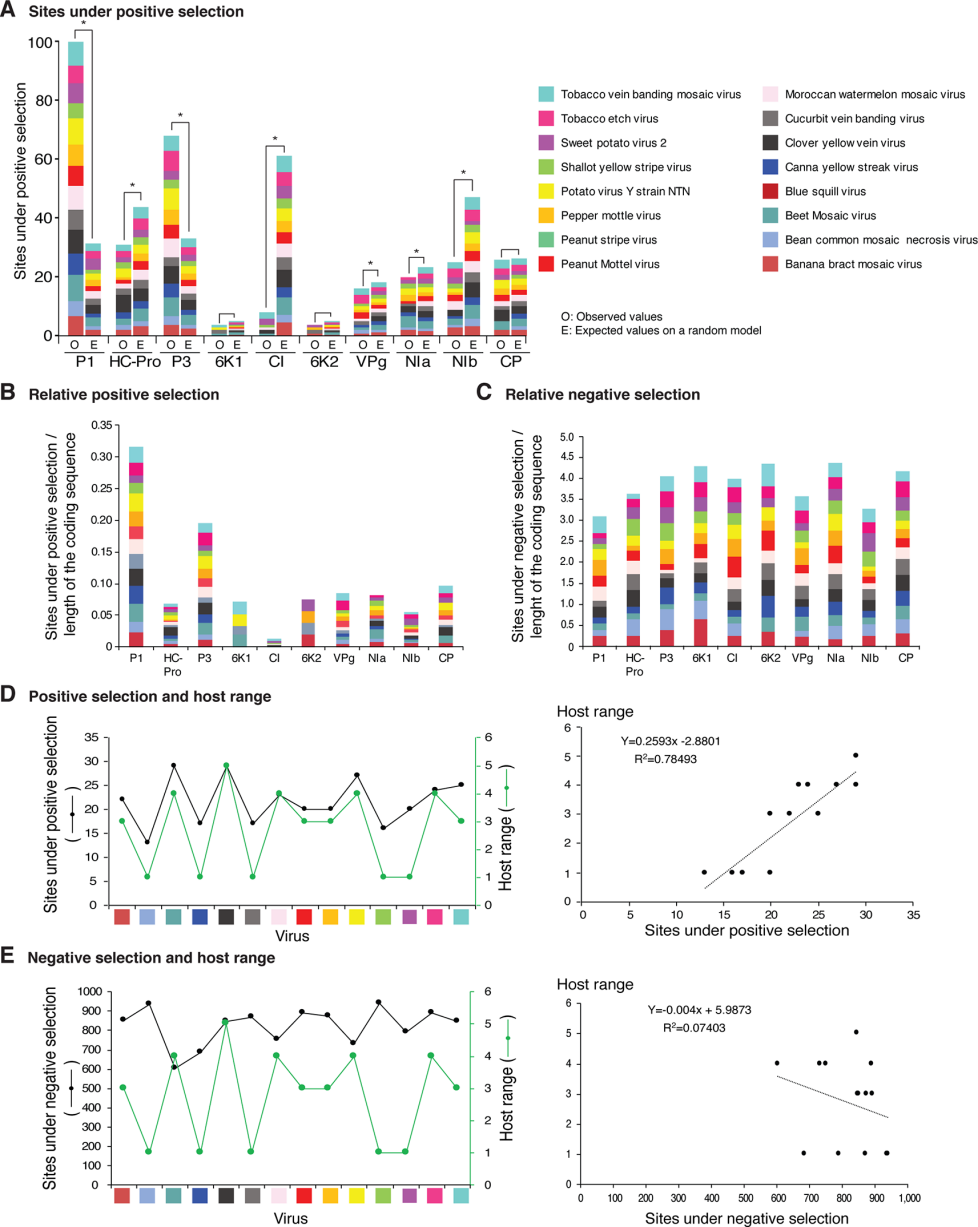
Frequency of sites under positive or negative selection per cistron and their relation with host range in the 16 viruses with the lowest detectable nucleotide and polyprotein variation. Labels are as in **Figure 12**.

Positive and negative selection sites were determined for the sixteen potyviruses with the highest (**Figure 12**) and lowest (**Figure 13**) variation index in nucleotide and amino acid sequence. Thirteen of the top sixteen viruses exhibited higher than average nucleotide variation for all potyviruses. In contrast, all sixteen least variable viruses exhibited lower than the average nucleotide variation (**Figure 1A**). The sixteen least variable viruses had ∼0.4 lower number of sites under positive or negative selection compared to the sixteen most variable viruses (**Figures 12A, B** and **13A, B**).

Per cistron, the abundance of sites under negative selection was approximately 10-fold (**Figures 12B, C** and **Supplementary Table S4**) and 14-fold higher (**Figures 13B, C**) than sites under positive selection for the sixteen most and least variable viruses, respectively. However, per cistron, the accumulation of sites under positive or negative selection followed similar patterns. Accordingly, these differences are not determined by the number of accessions.

The majority of sites in the potyviral genome are under negative selection (**Figures 12** and **13**). A small number of sites under positive selection were identified (dN/dS ratio > 5, p-value ≤0.05 ; **Supplementary Figure S8**). After normalizing for the length of each cistron, the number of sites under positive selection was higher than the expected randomly in P1, P3, CP, and NIa. In all other cistrons, the number of sites under positive selection was lower than would be expected randomly (**Figures 12A** and **13A**). Cistrons containing hypervariable areas in the potyviral genome also harbored the highest frequency of sites under positive selection. P1 contained the highest number of positive selection sites. In contrast, CI had the fewest number of sites under positive selection (**Figures 12A** and **13A**). This implies that P1 is the most genetically variable cistron, while CI is the most stable cistron. In the CP, the number of sites under positive selection was higher than expected only for the 16 most variable potyviruses (**Figures 12A** and **13A**).

### Sites Under Positive Selection and Host Range

Different host species impose heterogeneous selective constraints and select for genetic variants with a competitive advantage (Longdon et al., 2014; Huang et al., 2015). This model predicts that viruses with a wide host range have more sites under positive selection than viruses with a narrow host range. To test this hypothesis, we plotted the number of host plants and the number of sites under positive selection for the sixteen most variable viruses and for the sixteen viruses with the least detectable variation. Both variables were also plotted against the number of accessions. A linear correlation was obtained between the number of sites under positive selection and host range (**Figures 12D** and **13D**). No correlation was observed between the number of sites under negative selection and host range (**Figure 13E** and **Supplementary Figure S14**). Collectively and individually, the sixteen most variable potyviruses had a linear correlation between the number of accessions and the number of sites under positive selection and the number of accessions and the host range (**Figure 12E**). The exception was WMV because the number of sites under positive selection reached a saturation point.

### Nucleotide Substitution Are Biased Towards Transitions

Twelve nucleotide substitutions are possible and can be divided into transitions (purine to purine or pyrimidine to pyrimidine changes) and transversions (purine to pyrimidine changes). If the occurrence is random, transversions (A↔C, A↔U, G↔C, and G↔U) should occur twice as often as transitions (A↔G and U↔C), (Lyons and Lauring, 2017). Thus, transitions (each at 8.3%) and transversions (each at 8.3%) are expected to account for 33.2% and 66.8% of the nucleotide substitutions, respectively. For the 79 potyviral species, transitions and transversions accounted for 71% and 29% of the nucleotide substitutions, respectively (**Supplementary Figure S9A**). The transversion frequency was 2.3-fold lower (p-value ≤ 0.00001) and the frequency of transitions was 2.1-fold higher (p-value ≤0.00001) than would be expected randomly. A to G (19.0%) and T to C (18.7%) transitions were the most frequent. Substitutions in the opposite direction, C to T (16.9%) and G to A (16.8%), were less frequent (**Supplementary Figure S9**). Thus, A to G, and T to C transitions were 2.3-fold higher (p-value ≤ 0.0001) than C to T and G to A substitutions. Although nucleotide substitutions may occur randomly, they do not accumulate in a random way. Instead selection results in a net gain in GC content and suggest that nucleotide substitutions preferentially accumulate in areas with low GC content. Variation detected in available sequences represent only nucleotide substitutions that result in functional changes, possibly conferring a selective advantage.

We performed a two-way GC content analysis to determine the relationship between nucleotide substitutions and GC content. Areas with high or low GC content are not conserved across potyvirus genomes. Clusters formed by viral species and by areas of the genome (**Supplementary Figure S10**) did not correlate with SNP clusters nor with hypervariable areas (**Supplementary Figure S4**). To eliminate the effect of low variation, the analysis was limited to the 21 species with high genomic variation. Distribution and abundance of nucleotide substitutions did not correlate with GC content. For potyviruses, nucleotide substitutions are biased towards transitions and result in a net gain in GC content. However, GC content does not determine their distribution in the genome. This is consistent with the model that nucleotide composition and translational selection do not explain codon usage in plant viruses (Cardinale et al., 2013).

### Amino Acid Substitutions Are Not Determined by Codon Usage

Viral proteins are multifunctional, functional diversity is mediated by structural flexibility, and amino acids promote either order or disorder in proteins (Campen et al., 2008; Rantalainen et al., 2011). Using the consensus sequence of each species, we determined relative synonymous Codon Usage (RSCU) in the 79 potyvirus species containing nucleotide substitutions (**Supplementary Table S5**). For the 38 most abundant codons, 26 end in A/G (fourteen end in A and 12 end in G) and the remaining twelve end in U/C. To determine if codon usage is related to the genomic distribution of nucleotide substitutions, we performed a two-way cluster analysis. Three clusters were observed (**Supplementary Figure S11**). Over-represented codons ending in A or G formed a large cluster. The 38 underrepresented codons ending in C or U formed another cluster. The final cluster was formed by those between the first two. In the polyprotein analysis, SCMV, PPV and bean yellow mosaic virus (BYMV) formed a cluster (**Supplementary Figure S6**). Codon usage placed these viruses in different clusters (**Supplementary Figure S11**). Contrasting clusters were observed for other species. Accordingly, potyviruses maintain preference for codons ending in A or G while codons ending in C or U occur at low frequency, and amino acid substitutions are not related to codon usage.

### Amino Acid Substitution Profile

We determined the amino acid content and profiled amino acid substitutions to examine the effect of genomic variation on polyprotein variation. The most abundant amino acids were Leu (8.8%), Lys (7.2%), Val (6.7%), Glu (6.7%), Ser (6.6%), Ala (6.4%), Thr (6.1%), Ile (6.0%), Gly (5.9%), Asp (5.3%), Arg (5.3%). Each other amino acid accounted for less than 5% (**Supplementary Table S6** and **Supplementary Figure S12A**). The four most frequent amino acid substitutions were Lys to Arg, Arg to Lys, Val to Ile and Ile to Val. They accounted for 71% of the events (**Supplementary Figure S12B**). Lys to Arg were more abundant than Arg to Lys substitutions (1.6 fold, **Supplementary Figure S12C**). In contrast, Val to Ile were equally abundant as Ile to Val substitutions. Arginine promotes disorder in proteins (Campen et al., 2008). In contrast, Tryptophan promotes protein stability (Campen et al., 2008) and was the least abundant amino acid (**Supplementary Figure S12A**). Thus, in potyviruses, amino acid substitutions favor arginine enrichment and disorder in proteins.

### Variation At the NIb-CP Junction

Our two-way cluster analysis of nucleotide and polyprotein variation (**Supplementary Figures S4** and **S6**) showed hypervariation at the NIb-CP junction. Our genome-wide and polyprotein-wide variation analyses identified a hypervariable area at the NIb-CP junction in the sixteen potyviruses with the most nucleotide diversity (**Figures 2** **–** **11**). This is consistent with the high proportion of sites under positive selection in the CP (**Figure 12A**).

After P1, the NIb-CP junction harbors the most variation for the most variable potyviruses (**Figures 2** **–** **11**). This observation is in agreement with a recent study showing that based on variation at the NIb-CP junction SCMV in Kenya consists of at least three strains. Variation maps to four discrete areas containing insertions, deletions, and nucleotide substitutions. Zone I is located within the last 33 amino acids of NIb, upstream of the NIa cleavage site. Zones II to IV are within the first 52 amino acids at the N terminal part of the CP (**Figure 2C**). Variation at the NIb-CP junction could be related to host, geographical origin, or both. To test this model, a SCMV phylogenetic tree was generated using the 91 genomic sequences from GenBank and six accessions from Kenya (Wamaitha et al., 2018). This data set represented ten countries and three hosts. Phylogeny, variation at the NIb-CP junction, geographical origin, and host were plotted in the same figure. Accessions from maize and sugarcane formed separate clusters regardless of the geographical origin. Accessions from sugarcane contained insertions in zones I and II. These insertions were not detected in accessions from maize, which contain insertions in zones I and IV regardless of the geographical origin (**Figure 2D**). Accordingly, variation at NIb-CP correlated with the host, regardless of the geographical origin.

Similar models were obtained for other potyviruses (**Figures 3**–**11**). Insertions or deletions at the N terminal part of the CP were identified in dasheen mosaic virus (DMV) (**Figure 3C**), SMV (**Figure 4C**), PPV (**Figure 5C**), PRSV (**Figure 6C**), and watermelon mosaic virus (WMV) (**Figure 7C**). Nucleotide substitutions at the NIb-CP junction were detected in other viruses (**Figures 8** **–** **11**). Accessions with insertion or deletions clustered separately and variation at the NIb-CP junction correlated with the host and country of origin for SMV (**Figure 4C**), PPV (**Figure 5C**), PRSV (**Figure 6C**), and WMV (**Figure 7C**). In DMV (**Figure 3C**), ZYMV (**Figure 8C**), BYMV (**Figure 9C**), TuMV (**Figure 10C**), and PVY (**Figure 11C**). Collectively, these results show that hypervariation at the NIb-CP junction is a general feature of potyviruses.

### Coat Protein Variation

**Figure 14:**
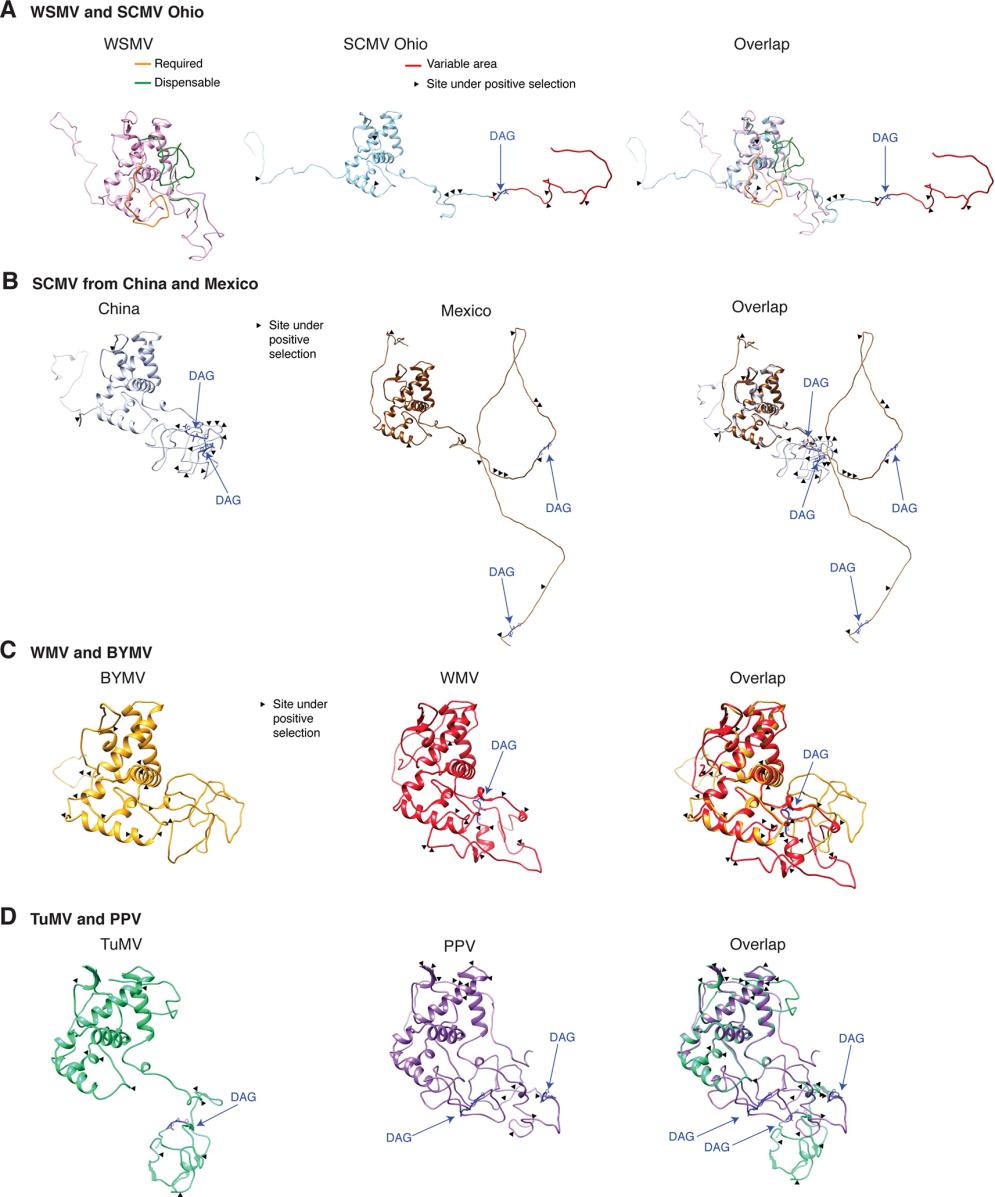
Schematic representation of the coat protein structural model. For potyviruses with the most amino acid variation, models were generated and superimposed using Phyre2 and Chimera v1.13, respectively. Wheat streak mosaic (WSMV; NC_001886) was used as a control. The coat protein folds into a central core, an N terminal and a C terminal variable loops. TM score and RMSD for each of the analyzed pairs were 0.49 ± 0.60 and 2.0 ± 2.4, respectively. Positive selection sites and the DAG motif are indicated. **(A)** Model for the Ohio isolate (AFQ35988.1) of SCMV compared to WSMV. **(B)** Representative SCMV isolates from China (AGE32037.1) and Mexico (ADG23201.1). **(C)** Bean yellow mosaic virus (NP_612218.1) and watermelon mosaic virus (ABD59007.1). **(D)** TuMV (NP_062866.2) and PPV (AFJ74692.1).

**Figure 15:**
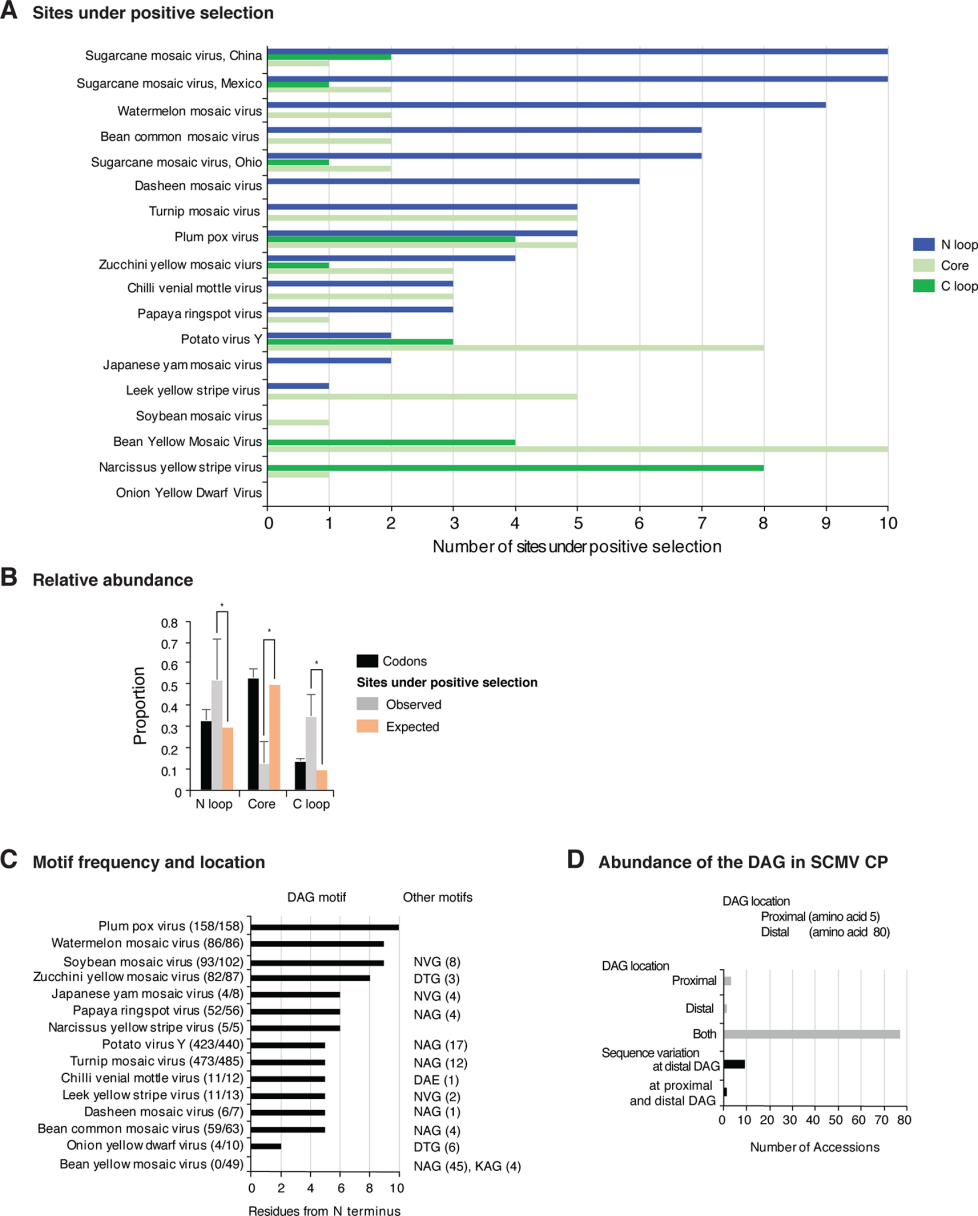
Variation in the coat protein and DAG motif. **(A)** Frequency of sites under positive selection in the N terminal loop, core domain, and C terminal loop for the top 16 most variable viruses. Three isolates of SCMV are represented. Sites under positive selection in each part of the coat protein were determined as in **Figure 6** and mapped using NCBI graphic format. **(B)** Abundance of sites under positive selection relative to the number of codons in each part of the coat protein. Bars represent average (± standard error) of the species indicated in panel **(A)**. *indicates significant differences with p-value ≤ 0.001 as determined by the Chi-square test. **(C)** Frequency of the DAG motif and distance from the N terminus. Numbers in parenthesis indicate accessions with the DAG motif and the total number of accessions. The frequency of other abundant motifs is indicated. **(D)** Abundance and variation in location of the proximal and distal DAG motifs in the SCMV.

Variation at the N terminus of the CP may affect protein organization and topology. To test this hypothesis, the CP of three diverse SMCV isolates was subjected to a structural alignment. The Ohio isolate (AFQ35988.1 from maize) was used as reference and compared to a maize isolate from China (AGE32037.1) and Mexico (ADG23201.1). The WSMV CP was used as a control. The model showed that the CP forms a core domain, an N-terminal flexible loop, and a C terminal flexible loop (**Figure 14A**). Similar models were obtained for other potyviruses (**Figure 14**). The core domain aligned across species and isolates. However, the N and C terminal loops were variable. The hypervariable area of the CP mapped to N terminal loop outside of the core domain. In our analysis (**Figures 14** and **15**), the N-terminal part contained the highest proportion of nucleotide substitutions as well as the Asp-Ala-Gly (DAG) motif required for aphid transmission (Lopez-Moya et al., 1999).

The largest number of sites under positive selection in the CP was obtained for BYMV, PPV, LYSV, and WMV (**Figure 12A**). In BYMV, 10 of 14 sites under positive selection mapped to the core domain. In all other viruses analyzed, the hypervariable area and most of the sites under positive selection mapped to the N terminal loop (**Figures 15A, B**). After normalizing for the length of each region, the variable N and C terminal loops harbor more variation and the core less variation than would be expected to occur through random chance (p-value ≤0.005) (**Figure 15B**).

The N and C terminal variable loops may interact with host or vector factors and participate in host adaptation, vector transmission, or both. Consistent with this model, our analysis identified a hypervariable area at the N terminal part of WSMV (**Figure 14A** and **Supplementary Figure S13**). Interestingly, mutants lacking amino acids that form part of the hypervariable area cause more severe symptoms than the wild type virus (Tatineni et al., 2017). Furthermore, our analysis identified a hypervariable area at the N terminal part of the PVY CP (**Figure 11A**). This area contains two sites under positive selection that affect virus accumulation in tobacco and potato. One of these sites also affects aphid transmission (Moury and Simon, 2011).

### Variation at the Motif Required for Aphid Transmission

Mutational analyses with several potyviruses showed that the CP contains a DAG motif which, along with surrounding amino acids, interacts with HC-Pro to mediate aphid transmission (Lopez-Moya et al., 1999; Dombrovsky et al., 2005). Although the DAG motif is believed to be highly conserved (Harrison and Robinson, 1988; Atreya et al., 1995), aphid transmission is mediated by a combination of amino acids in the N terminus not only by the DAG motif (Dombrovsky et al., 2005). We profiled the amino acid composition and location of the DAG motif in the 16 most variable potyviruses. Results show that there is variation in both amino acid composition and distance from the N terminus. The most frequent deviations from DAG were NAG and NVG (**Figure 15C**). Emphasizing this result, the BYMV CP did not contain a DAG motif. In SCMV, 77 of the 91 accessions analyzed contain two DAG motifs, one at position 5 (proximal) and one at position 80 (distal) from the N terminus. Out of the other 14 accessions, 9 have variation in the position of the distal DAG motif and one had variation in the position of both motifs. Three accessions contained only the proximal and one accession contained only the distal DAG motif (**Figure 15D**). Accordingly, the DAG motif is not universally conserved. There is variation both in sequence and location.

## Discussion

Potyviruses represent one-quarter of known plant RNA viruses (Gibbs et al., 2008; Gibbs and Ohshima, 2010). Currently, 2,026 plant species from 556 genera and 81 botanical families, distributed world-wide, are susceptible to potyviruses (Rivett et al., 1996). Potyviruses are transmitted in a non-persistent way by more than 200 species of aphids (Shukla et al., 1994; Gibbs et al., 2008). This is an indication that potyviruses have an outstanding capacity to adapt to new hosts, vectors, and environments. In this study, genome-wide and polyprotein-wide variation analyses showed that the potyviral genome contains hypervariable areas that preferentially accumulate nucleotide substitutions, sites under positive selection (**Figures 2** **–** **11**, **12A**, and **13A**), promote disorder in proteins and may be determinants of host adaptation (**Supplementary Figure S15**).

### Evolutionary Constraints on Potyviruses

Host and vector factors, the environment, and their interactions impose external evolutionary constraints (Lopez-Moya et al., 1999; Wylie et al., 2002; Huang et al., 2015; Willemsen et al., 2016).

Essential functions, such as RNA replication, virion formation, and movement are accomplished by viral factors working in synchrony with host factors. These complexes often require interactions between viral proteins, viral RNA, or both (Wan et al., 2015; Li et al., 2016). Emphasizing this point, multiple interactions have been described between potyviral proteins (Jiang and Laliberte, 2011; Revers and Garcia, 2015). Thus, functionality of viral RNA, proteins, and their interactions impose internal evolutionary constraints.

The endopeptidases P1, HC-Pro, and NIa-Pro process the polyproteins formed by translation of potyviral RNA (Revers and Garcia, 2015). P1 and HC-Pro catalyze their own separation from the polyprotein, and the remaining seven proteolytic sites are processed by NIa-Pro. Cleavage sites between individual proteins are specific for each protease and are not interchangeable (Adams et al., 2005a). Accordingly, the NIa-Pro catalytic domain and cleavage sites must maintain their identity, imposing an internal constrain on variation.

### Variation at NIa-Pro Cleavage Sites

Fourteen of the sixteen potyviruses with the most diversity harbor variation at the NIa-Pro cleavage site between NIb and CP. Consistent with this observation, variation was detected in the NIa-Pro cleavage sites of sweet potato mild mottle virus (Adams et al., 2005a), and SCMV from Kenya (Wamaitha et al., 2018). Accordingly, variation at this cleavage site might be a general feature of potyviruses. Variation at the NIb-CP junction could be an indication that polypeptide flexibility and flexible disorder (Tokuriki et al., 2009) are required for exposing the NIb-CP cleavage site to NIa-Pro and to the multiple functions of the CP. It may also suggest that the NIb-CP junction confers mutational robustness that allows generation of the diversity required for variants with a competitive advantage to be selected and promote virus adaptation. Additionally, the number of sites under positive selection in NIa-Pro is higher than expected randomly (**Figure 12A**), suggesting that the NIa-Pro catalytic domain is variable.

### Hypervariable Areas in the Potyviral Genome

Nucleotide substitutions preferentially accumulate at the 5'UTR, the N terminal part of P1, N and C terminal parts of HC-Pro, the C terminal part of P3, VPg, the C terminal part of NIb, and the N terminal part of the CP. The same cistrons were identified as hypervariable through SAP analysis (**Figures 2** **–** **11**, **Supplementary Figures S5** and **S7**). These observations are in agreement with a previous study showing that P1 and P3 exhibit higher variation than other proteins (Adams et al., 2005b).

In addition to SNPs and SAPs, the frequency of sites under negative or positive selection is not uniform across the potyviral genome (**Figures 2** **–** **11**, **Supplementary Figure S8**). However, genomic and polyprotein variation were consistent in the sixteen most variable (5 to 485 accessions) and the sixteen least variable (3 to 24 accession) potyviral groups (**Figures 12** ****and**13**). Negative selection sites were 10- to 14-fold more abundant than positive selection sites, indicating that the potyviral genome is under strong negative selection (**Figures 12** ****and **13**). The largest proportion of sites under negative selection was found in CI (**Figures 12** and **13**). Thus, CI is the most genetically stable cistron in the potyviral genome, as reported previously (Adams et al., 2005b). P1, P3, NIa-Pro, VPg and CP were the most variable and contained the highest proportion of sites under positive selection (**Figures 12** ****and **13**).

Abundance of sites under positive and negative selection classified potyviral proteins into two groups. 6K1, CI, 6K2, and NIb have the lowest frequency of sites under positive selection and the highest frequency of sites under negative selection (**Figures 12A–C** and **13A–C**). These proteins participate in the formation and movement of virus replication complexes, which require interaction with cellular membranes (Wan et al., 2015). In contrast, proteins with the highest frequency of positive selection sites and the lowest frequency of negative selection sites (P1, P3, NIa, and CP) participate in translation, polyprotein processing, virion formation, and vector transmission (Wei et al., 2010; Rantalainen et al., 2011; Ivanov et al., 2014). These functions require interactions with the cellular translational machinery (Ivanov et al., 2014; Pasin et al., 2014). Both HC-Pro and VPg contain hypervariable areas (**Figures 2** **–** **11**), are multifunctional proteins, interact with the cellular translational machinery, and require host factors to effectively suppress gene silencing (Ivanov et al., 2016; Cheng and Wang, 2017).

The genetic diversity of host and vector interaction partners may explain the differences in the positive and negative selection site accumulation. Specifically, these differences may be between proteins involved in formation and movement of virus replication complexes versus proteins involved in translation, virion formation, vector transmission, and silencing suppression.

### P1 Variation

Potyviral P1 protein participates in translation and is a modulator of RNA replication (Revers and Garcia, 2015). In PPV, the N terminal part of P1 is hypervariable, disordered, dispensable for virus replication, implicated in adaptation to new hosts, host defense responses, and host-dependent pathogenicity (Valli et al., 2007; Rohozkova and Navratil, 2011; Maliogka et al., 2012; Pasin et al., 2014). It is also a symptom determinant (Pasin et al., 2014). Our analysis showed that potyviral P1 is the most variable cistron, contains the highest proportion of sites under positive selection, and variation maps to the N terminal part (**Figure 14**). These observations suggest that potyviral P1 is a determinant of host adaptation.

### HC-Pro Variation

RNA silencing suppressors promote virus susceptibility by interfering with antiviral defense, show high sequence diversity, and contain residues under positive selection (Murray et al., 2013). HC-Pro is an essential gene silencing suppressor in potyviruses (Garcia-Ruiz et al., 2010; Revers and Garcia, 2015). The N and C terminal parts of HC-Pro are hypervariable (**Figures 2** **–** **4**). However, the number of sites under positive selection is lower than would be expected randomly (**Figures 12** and **13**). Substitutions in HC-Pro affect silencing suppression activity and pathogenicity (Torres-Barcelo et al., 2008). Thus, the low number of sites under positive selection could be explained by the inactivating effect of mutations in the central region, which is essential for silencing suppression (Garcia-Ruiz et al., 2010).

### P3 Variation

In SMV, a single amino acid change in P3 resulted in increased pathogenicity in soybean cultivars (Wen et al., 2011). In our analysis, the C terminal part of P3 is hypervariable, and P3 is the cistron with the second highest number of sites under positive selection (**Figures 12A, B**). This suggests that P3 is a determinant of pathogenicity and host adaptation.

### VPg Variation

Potyviral VPg is intrinsically disordered, a property that mediates functional diversity and interactions with multiple partners (Hebrard et al., 2009; Jiang and Laliberte, 2011; Rantalainen et al., 2011; Cheng and Wang, 2017). This is consistent with VPg participation in viral RNA translation, silencing suppression, RNA replication, cell-to-cell and systemic virus movement (Revers and Garcia, 2015; Cheng and Wang, 2017). Each of these roles is mediated by different host factors (Cheng and Wang, 2017). For PVY VPg, structural flexibility is associated with host adaptation (Charon et al., 2018). Our analysis found that, for several species, including SCMV, WMV and PVY, VPg is hypervariable and accumulates more sites under positive selection than randomly expected (**Figures 12A** and **13A**). These observations suggest a role for potyviral VPg in host adaptation.

### NIb Variation

NIb codes for the RNA-dependent-RNA polymerase responsible for RNA replication (Revers and Garcia, 2015). Variation in NIb localizes to the C terminal part near the junction with the CP. However, for the entire cistron, the number of sites under positive selection was less than would be expected randomly (**Figures 12A** and **13A**). Thus, NIb is under strong negative selection. However, variation at the NIb-CP junction might be related to efficiency of polyprotein processing by NIa-Pro.

### CP Variation

The CP participates in virion formation, cell-to-cell movement, and systemic movement (Ivanov et al., 2014). The N terminal end of the CP contains a conserved DAG motif, which interacts with HC-Pro to mediate aphid transmission (Lopez-Moya et al., 1999; Dombrovsky et al., 2005). Aphid transmissibility in potyviruses is lost after repeated mechanical passages (Wylie et al., 2002; Kehoe et al., 2014). Accordingly, there is selection pressure to conserve protein-protein interactions that mediate aphid transmissibility for any particular plant-virus-vector combination (Wylie et al., 2002). This model does not account for the possibility of a virus being delivered by a vector on a different host, nor for the possibility of different virus vectors. We propose that genetic flexibility in the CP and in the aphid-transmission motif are necessary to maintain functionality in genetically diverse hosts and vectors (**Supplementary Figure S15**).

Structural modeling (**Figure 14**), biochemical analysis (Shukla et al., 1988), tritium bombardment (Baratova et al., 2001), and physicochemical characterization (Ksenofontov et al., 2013) suggest that both the DAG motif and the hypervariable area map to the N terminal flexible loop. The biochemical analysis showed that the N- and a C-terminal parts of the CP are exposed on the surface of the virions and that a core protects the viral RNA. The exposed N-terminus consists of 30 to 69 amino acids, the exposed C terminal 17 to 20 amino acids, and the core 216 to 218 amino acids (Shukla et al., 1988). The core domain is required for RNA encapsidation and virion formation (Revers and Garcia, 2015; Zamora et al., 2017). Cryo-electron microscopy analysis of the WMV CP showed that the N and C termini forms flexible arms and a core domain rich in alpha helices (Zamora et al., 2017).

Furthermore, the DAG motif is not universally conserved. There is variation in amino acid sequence and in location of the DAG motif (**Figures 15C, D**). In eleven of fifteen other viruses analyzed, NAG, NVG or DTG frequently replaced the DAG motif (**Figure 15C**). BYMV lacks a DAG motif and contains a NAG or a KAG motif instead. Interestingly, BYMV exhibits high genetic variation (**Figures 1** and **9**), has a wide host range that includes 200 plant species in 14 families, and is transmitted by over 50 aphid species (Kehoe et al., 2014). Consistent with these results, several potyviruses that are transmitted by aphids do not contain a DAG motif (Johansen et al., 1996; Wylie et al., 2002). Vector specificity may be explained by structural complementation between the N terminus of the CP and the hinge domain in HC-Pro (Dombrovsky et al., 2005). Consistent with the flexible disorder observed in some RNA virus proteins (Tokuriki et al., 2009), these observations suggest a correlation between variation in CP, aphid-transmission, and virus-vector specificity.

Essential CP functions likely require interaction with different host or vector factors (Garcia-Ruiz, 2018). Flexible disorder (Tokuriki et al., 2009) may contribute to the multiple functions of the CP in a genetically diverse array of vectors and hosts. Variation at the N terminal part of PPV CP determines host-dependent pathogenicity (Carbonell et al., 2013), and sites under positive selection at the N terminal part of the PVY CP affect fitness in tobacco and potato (Moury and Simon, 2011). Characterization of PVA supports a model in which the CP harbors disorder structures, while the virion structure is stable. CP disorder may be needed for multiple biological functions (Baratova et al., 2001; Ksenofontov et al., 2013). Furthermore, systemic movement of tobacco etch virus (TEV), lettuce mosaic virus (LMV), and some isolates of PPV is restricted by RTM1, RTM2, and RTM3 (Decroocq et al., 2009), which are expressed in the phloem sieve elements and interact with the viral CP (Chisholm et al., 2001). Interestingly, TEV resistance braking isolates emerged though mutations in the N-terminus of the CP (Decroocq et al., 2009). Collectively, these observations support a role for the hypervariable area at the N terminus of the CP in host adaptation.

### Hypervariable Areas as Determinants of Host Adaptation

Intrinsically disordered proteins are over represented in viruses and participate in processes that require interactions between host and viral proteins (Borkosky et al., 2017). Disorder in proteins provides multiple advantages such as interacting with and adapting to multiple conformations when binding with different partners, which allows participation in signaling or regulatory events (Babu, 2016; Borkosky et al., 2017). Consistent with this model, proteins P1, VPg, and the CP are structurally disordered (Charon et al., 2016), interact with multiple host factors (Shi et al., 2007; Pasin et al., 2014; Cheng and Wang, 2017), and, in some potyviruses, P1, P3, VPg, and CP contribute to host adaptation (Revers and Garcia, 2015; Charon et al., 2018). Our analysis showed that P1, P3, VPg, and the CP contain hypervariable areas and sites under positive selection (**Figure 12A**), and suggest that genetic flexibility in key parts of the potyviral genome is necessary to maintain functionality in genetically diverse hosts and vectors, an in diverse environments. We propose that fixed hypervariable areas in the potyviral genome are determinants of host adaptation (**Supplementary Figure S15**).

### Variation in the Number of Sequences Analyzed

The relationship between the abundance of SNPs or SAPs and the number of accessions analyzed follows a rarefaction curve (Chiarucci et al., 2009) and can be explained by a logarithmic function with polymorphisms reaching a saturation point (**Figures 1C, D** and **Supplementary Figure S3**). Genomic and polyprotein variation indexes are not adjusted for the number of accessions. However, the nucleotide diversity (Pi) index (**Figure 1A**), uses a correction factor for the number of accessions (Rozas, 2009). For each virus species, genome-wide distribution of SNPs, nucleotide diversity, and SAPs, all independently point to common hypervariable areas (**Figures 2** **–** **11** and **Supplementary Figures S5** and **S7**). Accordingly, the distribution of hypervariable areas in the potyviral genome is unlikely to be biased by the number of accessions. This is illustrated by the comparison between DMV (**Figure 3**) and TuMV (**Figure 10**). For DMV and TuMV, genomic variation was profiled using 7 and 483 nucleotide accessions, respectively (**Figure 1A**). For both viruses, hypervariable areas were identified at the N-terminal part of P1 and at the NIb-CP junction (**Figures 3A** and **10A**). Additionally, both areas were also identified as hypervariable using 7 or 485 polyprotein sequences (**Figures 3B** and **10B**).

In the sixteen most (**Figure 12A**) and sixteen least variable viruses (**Figure 13A**), sites under positive selection accumulated to higher frequency in P1, P3, and CP, while CI accumulated the lowest frequency. These two groups of viruses, have contrasting number of accessions (**Figure 1A**). Accordingly, the distribution of sites under positive selection in the potyviral genome is unlikely to be biased by the number of accessions.

Consistent with a rarefaction curve, a saturation pattern in nucleotide substitutions is reached for potyviruses in general (**Figure 1C** and **Supplementary Figure S3A**) and, specifically, for each of the most variable species (**Figure 1D** and **Supplementary Figure S3B**). In contrast, a linear correlation was observed between the number of sites under positive selection and host range, and between the number of accessions, sites under positive selection, and host range for the top 16 most variable potyviruses (**Figures 12D, E**). For TuMV, the number of hosts increased linearly from 10 to 438 accessions (**Figure 12E**), while the number of SNPs reached a saturation point at 50 accessions (**Figure 1D**). A similar pattern was observed for other viruses (**Figures 1D** and **12E**).

In the most variable viruses a linear correlation was observed between the number of sites under positive selection and number of accessions (**Figure 12E**) and between the number of hosts and the number of accessions (**Figure 12D**). These viruses are represented by contrasting number of accessions and were analyzed individually. Accordingly, the relationship between the number of sites under positive selection, number of hosts, and number of accessions is unlikely to be biased by the number of accessions.

A model in which variation occurs in hypervariable areas and heterogeneous hosts impose diverse selection pressure, predicts that nucleotide substitutions will reach a saturation point, while the number of hosts, and sites under positive selection will increase with the number of accessions. Theoretically, a single nucleotide position in the genome can only account for one SNP. A codon can account for three SNPs and for one site under positive selection. However, a codon allows for multiple synonymous and non-synonymous substitutions. The dN/dS ratio used to identify sites under positive selection was estimated at each codon in the polyprotein open reading frame and requires multiple variants to reach a significance threshold (Rozas, 2009). Accordingly, adding more accessions may increase the number of hosts and the number of sites under positive selection without adding SNPs. These features of the model might link the number of hosts and the number of sites under positive selection in viruses with heterogeneous hosts, as observed here (**Figure 12E**). In contrasts, no link might be established when accessions come from the same hosts, as observed for WSMV (**Supplementary Figure S13D**) or when viruses are not under positive selection pressure.

These observations suggest that best way to identify the relationship between hosts and sites under positive selection is by using viruses with the highest number of accessions, the widest host range, and the highest genomic variation, as presented in **Figure 12**. Hypervariable areas identified in this study provide the foundation for the biological identification and characterization of viral, host, and vector factors that mediate host adaptation in potyviruses.

### A Model for Host Adaptation

Viruses co-evolve with their hosts and vectors in diverse environments. Accordingly, for any particular plant-virus-vector combination, viruses face selection pressure to conserve RNA and protein functions that mediate aphid transmissibility, replication and movement (Wylie et al., 2002). While hosts select for variants adapted to replication and movement, vectors select based on transmission efficiency before or after virus adaptation to a particular host (**Supplementary Figure S15**). Vector transmission before host adaptation may result in virus spread to diverse hosts. After host adaptation has been achieved, vector transmission could be restricted to a plant species and closely related relatives. Thus, genetic flexibility in key parts of the genome is necessary to maintain functionality in genetically diverse hosts, vectors, and environments. Tolerance for mutations at hypervariable areas may provide robustness necessary for the generation of selection diversity to identify variants with a competitive advantage. Repeated cycles of replication in a plant, vector transmission, and the corresponding selection may lead to host adaptation. In this model, hypervariation occurs in the cistrons that are viral determinants of host adaptation and vector transmission (**Supplementary Figure S15**).

This model may apply to other viruses. The five conserved cistrons of citrus tristeza virus are involved in replication and virion assembly. In contrast, the three variable cistrons are involved in host adaption and are determinants of host range (Tatineni et al., 2011). WSMV exhibits variation and tolerates deletions at the N terminal part of the CP. WSMV mutants lacking part of the N terminal have an expanded host range and cause more severe symptoms compared to wild type (Tatineni et al., 2017). In our analysis, the dispensable N terminal part was identified as hypervariable (**Supplementary Figure S13**). VPg and CP contain residues under positive selection in wheat yellow mosaic virus, (Sun et al., 2013), and in rice yellow mottle virus, and positive selection sites within P1 modulate the RNA silencing suppression activity (Sereme et al., 2014).

### Implications for Potyvirus Diagnostics and Plant Breeding

Potyviruses are detected through protein-dependent or RNA-dependent assays. Antibodies raised against virions are routinely used in ELISA tests or western blotting. Based on the assumption that the CP is stable, RT-PCR primers are often designed to target the N terminal part of the CP (Mahuku et al., 2015). For detecting SCMV in several African countries, ELISA and RT-PCR procedures have provided inconsistent results even in symptomatic tissue (Wamaitha et al., 2018).

Results described here provide an explanation and suggest an alternative. Antibodies were developed against, and primers were designed based on the Ohio isolate. However, in Kenya, SCMV consists of at least three genetic variants. Only one of them is similar to the Ohio isolate (Wamaitha et al., 2018). Thus, variation in the N terminal part of the CP was the reason for the erratic diagnosis of SCMV in Kenya.

For polyclonal antibodies raised against potyviruses, the epitope is derived from the N terminus (Shukla et al., 1988). Our results show that the N terminal part of the CP is hypervariable. Accordingly, an antibody raised against, and primers designed based on a particular isolate, are not likely to detect a genetic variant of the same or different strain. In contrast to CP, CI is the most genetically stable cistron in potyviruses (**Figure 12A**) (Adams et al., 2005b) so is therefore a better target to design RT-PCR primers for diagnostic purposes.

Large efforts are underway to develop maize cultivars tolerant or resistant to SCMV or maize lethal necrosis (Gowda et al., 2015). Given the fact that potyviruses harbor hypervariable areas and host plants select for the fittest variants, it is safe to predict that tolerance and resistance to SCMV or maize lethal necrosis will be broken by new strains. Those strains may not be as fit for hosts other than maize. Plant breeding efforts need to take into account the capacity of viruses to mutate and which areas are most likely to change. Perhaps, a geographical or temporal combination of susceptible and resistant cultivars or crops could be implemented to slow or prevent the emergence of resistance-breaking strains.

## Data Availability Statement

All datasets for this study are included in the article/**Supplementary Material**.

## Author Contributions

HG-R conceived the study. DN, KL, and HG-R performed the analysis. HG-R, DN, KL, and PS wrote the paper.

## Funding

This research was supported by NIH grant R01GM120108 to HG-R and by the Nebraska Agricultural Experiment Station with funding from the Hatch Act (Accession Number 1007272) through the USDA National Institute of Food and Agriculture. Open access costs were provided by the same grant.

## Conflict of Interest

The authors declare that the research was conducted in the absence of any commercial or financial relationships that could be construed as a potential conflict of interest.

## Supplementary Material

Supplementary Table S1 Nucleotide and polyprotein features of Potyviruses used in this study.

Supplementary Table S2 Purine vs. Pyrimidine ratio for the 95 Potyvirus genomes included in the genomic variation study.

Supplementary Table S3 Single Nucelotide Polymorphism (SNPs) by 50-nt bins for 79 Potyviruses with three or more accessions.

Supplementary Table S4 Number of sites under episodic positively selection as reported by SLAC (pervasive) and MEME (episodic) for ten cistrons in a selected group of potyviruses.

Supplementary Table S5 Codon Usage Bias for 79 Potyviruses with three or more accessions.

Supplementary Table S6 Amino acid profile of Polyproteins for Potyviruses in the genomic variation study.

## References

[B1] Abdel Azim G. Ben Othman M. Aboeleneen Z. (2011). Modified progressive strategy for multiple proteins sequence alignment. Int. J. Comput. 5 (Issue 1), 270–280.

[B2] Adams M. J. Antoniw J. F. Beaudoin F. (2005a). Overview and analysis of the polyprotein cleavage sites in the family Potyviridae. Mol. Plant Pathol. 6 (4), 471–487. 10.1111/j.1364-3703.2005.00296.x 20565672

[B3] Adams M. J. Antoniw J. F. Fauquet C. M. (2005b). Molecular criteria for genus and species discrimination within the family Potyviridae. Arch. Virol. 150 (3), 459–479. 10.1007/s00705-004-0440-6 15592889

[B4] Aryal R. Yang X. Yu Q. Sunkar R. Li L. Ming R. (2012). Asymmetric purine-pyrimidine distribution in cellular small RNA population of papaya. BMC Genomics 13 (1), 682. 10.1186/1471-2164-13-682 23216749 PMC3582581

[B5] Asnicar F. Weingart G. Tickle T. L. Huttenhower C. Segata N. (2015). Compact graphical representation of phylogenetic data and metadata with GraPhlAn. PeerJ 3, e1029. 10.7717/peerj.1029 26157614 PMC4476132

[B6] Atreya P. L. Lopez-Moya J. Chu M. Atreya C. D. Pirone T. P. (1995). Mutational analysis of the coat protein N-terminal amino acids involved in potyvirus transmission by aphids. J. Gen. Virol. 76 (2), 265–270. 10.1099/0022-1317-76-2-265 7844549

[B7] Babu M. M. (2016). The contribution of intrinsically disordered regions to protein function, cellular complexity, and human disease. Biochem. Soc. Trans. 44 (5), 1185–1200. 10.1042/bst20160172 27911701 PMC5095923

[B8] Baratova L. A. Efimov A. V. Dobrov E. N. Fedorova N. V. Hunt R. Badun G. A. (2001). *In situ *spatial organization of Potato virus A coat protein subunits as assessed by tritium bombardment. J. Virol. 75 (20), 9696–9702. 10.1128/JVI.75.20.9696-9702.2001 11559802 PMC114541

[B9] Bedhomme S. Lafforgue G. Elena S. F. (2012). Multihost experimental evolution of a plant RNA virus reveals local adaptation and host-specific mutations. Mol. Biol. Evol. 29 (5), 1481–1492. 10.1093/molbev/msr314 22319146

[B10] Bera B. C. Virmani N. Kumar N. Anand T. Pavulraj S. Rash A. (2017). Genetic and codon usage bias analyses of polymerase genes of equine influenza virus and its relation to evolution. BMC Genomics 18 (1), 652. 10.1007/s11427-006-2002-5 28830350 PMC5568313

[B11] Borkosky S. S. Camporeale G. Chemes L. B. Risso M. Noval M. G. Sanchez I. E. (2017). Hidden structural codes in protein intrinsic disorder. Biochemistry 56 (41), 5560–5569. 10.1021/acs.biochem.7b00721 28952717

[B12] Campen A. Williams R. M. Brown C. J. Meng J. Uversky V. N. Dunker A. K. (2008). TOP-IDP-scale: a new amino acid scale measuring propensity for intrinsic disorder. Protein Pept. Lett. 15 (9), 956–963. 10.2174/092986608785849164 18991772 PMC2676888

[B13] Carbonell A. Maliogka V. I. Perez J. D. Salvador B. San Leon D. Garcia J. A. (2013). Diverse amino acid changes at specific positions in the N-Terminal region of the coat protein allow plum pox virus to adapt to new hosts. Mol. Plant-Microbe Interact. 26 (10), 1211–1224. 10.1094/Mpmi-04-13-0093-R 23745677

[B14] Cardinale D. J. DeRosa K. Duffy S. (2013). Base composition and translational selection are insufficient to explain codon usage bias in plant viruses. Viruses 5 (1), 162–181. 10.3390/v5010162 23322170 PMC3564115

[B15] Charon J. Barra A. Walter J. Millot P. Hebrard E. Moury B. (2018). First experimental assessment of protein intrinsic disorder involvement in an RNA virus natural adaptive process. Mol. Biol. Evol. 35 (1), 38–49. 10.1093/molbev/msx249 29029259 PMC5850501

[B16] Charon J. Theil S. Nicaise V. Michon T. (2016). Protein intrinsic disorder within the potyvirus genus: from proteome-wide analysis to functional annotation. Mol. Biosyst. 12 (2), 634–652. 10.1039/c5mb00677e 26699268

[B17] Cheng X. Wang A. (2017). The potyvirus silencing suppressor protein vpg mediates degradation of SGS3 *via* ubiquitination and autophagy pathways. J. Virol. 91 (1). 10.1128/JVI.01478-16 PMC5165207 27795417

[B18] Chiarucci A. Bacaro G. Rocchini D. Ricotta C. Palmer M. W. Scheiner S. M. (2009). Spatially constrained rarefaction: incorporating the autocorrelated structure of biological communities into sample-based rarefaction. Community Ecol. 10 (2), 209–214. 10.1556/ComEc.10.2009.2.11

[B19] Chisholm S. T. Parra M. A. Anderberg R. J. Carrington J. C. (2001). *Arabidopsis *RTM1 and RTM2 genes function in phloem to restrict long-distance movement of tobacco etch virus. Plant Physiol. 127 (4), 1667–1675. 10.1104/pp.010479 11743111 PMC133571

[B20] Csorba T. Kontra L. Burgyan J. (2015). Viral silencing suppressors: Tools forged to fine-tune host-pathogen coexistence. Virology, 479–480. 10.1016/j.virol.2015.02.02885-103. 25766638

[B21] Cui H. Wang A. (2016). Plum pox virus 6K1 protein is required for viral replication and targets the viral replication complex at the early stage of infection. J. Virol. 90 (10), 5119–5131. 10.1128/JVI.00024-16 26962227 PMC4859702

[B22] Danecek P. Auton A. Abecasis G. Albers C. A. Banks E. DePristo M. A. (2011). The variant call format and VCFtools. Bioinformatics 27 (15), 2156–2158. 10.1093/bioinformatics/btr330 21653522 PMC3137218

[B23] Decroocq V. Salvador B. Sicard O. Glasa M. Cosson P. Svanella-Dumas L. (2009). The determinant of potyvirus ability to overcome the RTM resistance of *Arabidopsis thaliana* maps to the N-terminal region of the coat protein. Mol. Plant Microbe Interact. 22 (10), 1302–1311. 10.1094/MPMI-22-10-1302 19737103

[B24] Dombrovsky A. Huet H. Chejanovsky N. Raccah B. (2005). Aphid transmission of a potyvirus depends on suitability of the helper component and the N terminus of the coat protein. Arch. Virol. 150 (2), 287–298. 10.1007/s00705-004-0407-7 15503223

[B25] Gao F. Zhang C.-T. (2006). GC-Profile: a web-based tool for visualizing and analyzing the variation of GC content in genomic sequences. Nucleic Acids Res. 34, W686–W691. 10.1093/nar/gkl040 16845098 PMC1538862

[B26] Garcia-Arenal F. Fraile A. Malpica J. M. (2001). Variability and genetic structure of plant virus populations. Annu. Rev. Phytopathol. 39 (1), 157–186. 10.1146/annurev.phyto.39.1.157 11701863

[B27] Garcia-Arenal F. Fraile A. Malpica J. M. (2003). Variation and evolution of plant virus populations. Int. Microbiol. 6 (4), 225–232. 10.1007/s10123-003-0142-z 13680390

[B28] Garcia-Ruiz H. (2018). Susceptibility genes to plant viruses. Viruses 10 (9), 484. 10.3390/v10090484 PMC6164914 30201857

[B29] Garcia-Ruiz H. Takeda A. Chapman E. J. Sullivan C. M. Fahlgren N. Brempelis K. J. (2010). *Arabidopsis *RNA-dependent RNA polymerases and dicer-like proteins in antiviral defense and small interfering RNA biogenesis during Turnip Mosaic Virus infection. Plant Cell 22 (2), 481–496. 10.1105/tpc.109.073056 20190077 PMC2845422

[B30] Gibbs A. Ohshima K. (2010). Potyviruses and the digital revolution. Annu. Rev. Phytopathol. 48, 205–223. 10.1146/annurev-phyto-073009-114404 20438367

[B31] Gibbs A. J. Ohshima K. Phillips M. J. Gibbs M. J. (2008). The prehistory of potyviruses: their initial radiation was during the dawn of agriculture. PloS One 3 (6), e2523. 10.1371/journal.pone.0002523 18575612 PMC2429970

[B32] Gowda M. Das B. Makumbi D. Babu R. Semagn K. Mahuku G. (2015). Genome-wide association and genomic prediction of resistance to maize lethal necrosis disease in tropical maize germplasm. Theor. Appl. Genet. 128 (10), 1957–1968. 10.1007/s00122-015-2559-0 26152570 PMC4572053

[B33] Harrison B. D. Robinson D. (1988). Molecular variation in vector-borne plant viruses: epidemiological significance. Philos. Trans. R. Soc. London. B Biol. Sci. 321 (1207), 447–462. 10.1098/rstb.1988.0102 2907152

[B34] Hazra A. (2017). Using the confidence interval confidently. J. thoracic Dis. 9 (10), 4125–4130. 10.21037/jtd.2017.09.14 PMC5723800 29268424

[B35] Hebrard E. Bessin Y. Michon T. Longhi S. Uversky V. N. Delalande F. (2009). Intrinsic disorder in viral proteins genome-linked: experimental and predictive analyses. Virol. J. 6, 23. 10.1186/1743-422X-6-23 19220875 PMC2649914

[B36] Huang L. Z. Li Z. F. Wu J. X. Xu Y. Yang X. L. Fan L. J. (2015). Analysis of genetic variation and diversity of Rice stripe virus populations through high-throughput sequencing. Front. Plant Sci. 6 (176). 10.3389/fpls.2015.00176 PMC4371650 25852724

[B37] Ivanov K. I. Eskelin K. Basic M. De S. Lohmus A. Varjosalo M. (2016). Molecular insights into the function of the viral RNA silencing suppressor HCPro. Plant J. 85 (1), 30–45. 10.1111/tpj.13088 26611351

[B38] Ivanov K. I. Eskelin K. Lohmus A. Makinen K. (2014). Molecular and cellular mechanisms underlying potyvirus infection. J. Gen. Virol. 95 (7), 1415–1429. 10.1099/vir.0.064220-0 24722679

[B39] Jiang J. Laliberte J. F. (2011). The genome-linked protein VPg of plant viruses-a protein with many partners. Curr. Opin. Virol. 1 (5), 347–354. 10.1016/j.coviro.2011.09.010 22440836

[B40] Johansen I. E. Keller K. E. Dougherty W. G. Hampton R. O. (1996). Biological and molecular properties of a pathotype P-1 and a pathotype P-4 isolate of pea seed-borne mosaic virus. J. Gen. Virol. 77 (6), 1329–1333. 10.1099/0022-1317-77-6-1329 8683223

[B41] Katoh K. Standley D. M. (2013). MAFFT multiple sequence alignment software version 7: improvements in performance and usability. Mol. Biol. Evol. 30 (4), 772–780. 10.1093/molbev/mst010 23329690 PMC3603318

[B42] Kehoe M. A. Coutts B. A. Buirchell B. J. Jones R. A. (2014). Split personality of a Potyvirus: to specialize or not to specialize? PloS One 9 (8), e105770. 10.1371/journal.pone.0105770 25148372 PMC4141833

[B43] Kelley L. A. Mezulis S. Yates C. M. Wass M. N. Sternberg M. J. (2015). The Phyre2 web portal for protein modeling, prediction and analysis. Nat. Protoc. 10 (6), 845–858. 10.1038/nprot.2015.053 25950237 PMC5298202

[B44] Ksenofontov A. L. Paalme V. Arutyunyan A. M. Semenyuk P. I. Fedorova N. V. Rumvolt R. (2013). Partially disordered structure in intravirus coat protein of potyvirus potato virus A. PloS One 8 (7), e67830. 10.1371/journal.pone.0067830 23844104 PMC3700898

[B45] Kumar M. Thakur V. Raghava G. P. (2008). COPid: composition based protein identification. In silico Biol. 8 (2), 121–128. 18928200

[B46] Lefort V. Longueville J.-E. Gascuel O. (2017). SMS: Smart model selection in PhyML. Mol. Biol. Evol. 34 (9), 2422–2424. 10.1093/molbev/msx149 28472384 PMC5850602

[B47] Li Y. Xiong R. Bernards M. Wang A. (2016). Recruitment of *Arabidopsis *RNA helicase AtRH9 to the viral replication complex by viral replicase to promote turnip mosaic virus replication. Sci. Rep. 6, 30297. 10.1038/srep30297 27456972 PMC4960543

[B48] Longdon B. Brockhurst M. A. Russell C. A. Welch J. J. Jiggins F. M. (2014). The evolution and genetics of virus host shifts. PloS Pathog. 10 (11), e1004395. 10.1371/journal.ppat.1004395 25375777 PMC4223060

[B49] Lopez-Moya J. J. Wang R. Y. Pirone T. P. (1999). Context of the coat protein DAG motif affects potyvirus transmissibility by aphids. J. Gen. Virol. 80 (12), 3281–3288. 10.1099/0022-1317-80-12-3281 10567662

[B50] Lyons D. M. Lauring A. S. (2017). Evidence for the selective basis of transition-to-transversion substitution bias in two RNA viruses. Mol. Biol. Evol. 34 (12), 3205–3215. 10.1093/molbev/msx251 29029187 PMC5850290

[B51] Mahuku G. Lockhart B. E. Wanjala B. Jones M. W. Kimunye J. N. Stewart L. R. (2015). Maize Lethal Necrosis (MLN), an emerging threat to maize-based food security in sub-saharan Africa. Phytopathology 105 (7), 956–965. 10.1094/PHYTO-12-14-0367-FI 25822185

[B52] Maliogka V. I. Salvador B. Carbonell A. Saenz P. Leon D. S. Oliveros J. C. (2012). Virus variants with differences in the P1 protein coexist in a Plum pox virus population and display particular host-dependent pathogenicity features. Mol. Plant Pathol. 13 (8), 877–886. 10.1111/j.1364-3703.2012.00796.x 22458641 PMC6638729

[B53] Martin D. P. Murrell B. Golden M. Khoosal A. Muhire B. (2015). RDP4: Detection and analysis of recombination patterns in virus genomes. Virus Evol. 1 (1), vev003. 10.1093/ve/vev003 27774277 PMC5014473

[B54] Moury B. Morel C. Johansen E. Jacquemond M. (2002). Evidence for diversifying selection in potato virus Y and in the coat protein of other potyviruses. J. Gen. Virol. 83 (Pt 10), 2563–2573. 10.1099/0022-1317-83-10-2563 12237440

[B55] Moury B. Simon V. (2011). dN/dS-Based methods detect positive selection linked to trade-offs between different fitness traits in the coat protein of potato virus Y. Mol. Biol. Evol. 28 (9), 2707–2717. 10.1093/molbev/msr105 21498601

[B56] Murray G. G. Kosakovsky Pond S. L. Obbard D. J. (2013). Suppressors of RNAi from plant viruses are subject to episodic positive selection. Proc. Biol. Sci. 280 (1765), 20130965. 10.1098/rspb.2013.0965 23804618 PMC3712444

[B57] Murrell B. Wertheim J. O. Moola S. Weighill T. Scheffler K. Kosakovsky Pond S. L. (2012). Detecting individual sites subject to episodic diversifying selection. PloS Genet. 8 (7), e1002764. 10.1371/journal.pgen.1002764 22807683 PMC3395634

[B58] Obenauer J. C. Denson J. Mehta P. K. Su X. Mukatira S. Finkelstein D. B. (2006). Large-scale sequence analysis of avian influenza isolates. Science 311 (5767), 1576. 10.1126/science.1121586 16439620

[B59] Page A. J. Taylor B. Delaney A. J. Soares J. Seemann T. Keane J. A. (2016). SNP-sites: rapid efficient extraction of SNPs from multi-FASTA alignments. Microbial. Genomics 2 (4). 10.1099/mgen.0.000056 PMC5320690 28348851

[B60] Pasin F. Simon-Mateo C. Garcia J. A. (2014). The hypervariable amino-terminus of P1 protease modulates potyviral replication and host defense responses. PloS Pathog. 10 (3), e1003985. 10.1371/journal.ppat.1003985 24603811 PMC3946448

[B61] Rambaut A. (2009). FigTree. http://tree.bio.ed.ac.uk/software/figtree version 1.3.1. Available from: . Accessed 13 January 2015.

[B62] Rantalainen K. I. Eskelin K. Tompa P. Mäkinen K. (2011). Structural flexibility allows the functional diversity of potyvirus genome-linked protein VPg. J. Virol. 85 (5), 2449–2457. 10.1128/JVI.02051-10 21177813 PMC3067799

[B63] Revers F. Garcia J. A. (2015). Molecular biology of potyviruses. Adv. Virus Res. 92, 101–199. 10.1016/bs.aivir.2014.11.006 25701887

[B64] Rivett D. E. Ward C. W. Beikin L. M. Ramshaw J. A. M. Wilshire J. F. K. (1996). Lennox Legacy: The History of the CSIRO Laboratory at 343 Royal Parade Parkville. Australia: CSIRO Publishing.

[B65] Rodamilans B. Shan H. Pasin F. García J. A. (2018). Plant viral proteases: Beyond the role of peptide cutters. Front. Plant Sci. 9. 10.3389/fpls.2018.00666 29868107 PMC5967125

[B66] Rohozkova J. Navratil M. (2011). P1 peptidase–a mysterious protein of family *Potyviridae*. J. Biosci. 36 (1), 189–200. 10.1007/s12038-011-9020-6 21451259

[B67] Roossinck M. J. (2003). Plant RNA virus evolution. Curr. Opin. Microbiol. 6 (4), 406–409. 10.1016/s1369-5274(03)00087-0 12941413

[B68] Rozas J. (2009). DNA sequence polymorphism analysis using DnaSP. Methods Mol. Biol. 537, 337–350. 10.1007/978-1-59745-251-9_17 19378153

[B69] Schneider W. L. Roossinck M. J. (2001). Genetic diversity in RNA virus quasispecies is controlled by host-virus interactions. J. Virol. 75 (14), 6566–6571. 10.1128/Jvi.75.14.6566-6571.2001 11413324 PMC114380

[B70] Sereme D. Lacombe S. Konate M. Bangratz M. Pinel-Galzi A. Fargette D. (2014). Sites under positive selection modulate the RNA silencing suppressor activity of rice yellow mottle virus movement protein P1 . J. Gen. Virol. 95 (1), 213–218. 10.1099/vir.0.057026-0 24092757

[B71] Shen Y. Wan Z. Coarfa C. Drabek R. Chen L. Ostrowski E. A. (2010). A SNP discovery method to assess variant allele probability from next-generation resequencing data. Genome Res. 20 (2), 273–280. 10.1101/gr.096388.109 20019143 PMC2813483

[B72] Shi Y. Chen J. Hong X. Chen J. Adams M. J. (2007). A potyvirus P1 protein interacts with the Rieske Fe/S protein of its host. Mol. Plant Pathol. 8 (6), 785–790. 10.1111/j.1364-3703.2007.00426.x 20507538

[B73] Shukla D. M. Strike P. L. Tracy S. H. Gough K. Ward C. (1988). The N and C Termini of the Coat Proteins of Potyviruses Are Surface-located and the N Terminus Contains the Major Virus-specific Epitopes. J. Gen. Virol. 69, 1497–1508. 10.1099/0022-1317-69-7-1497

[B74] Shukla D. D. Ward C. W. Brunt A. A. (1994). The Potyviridae. (Wallingford, UK: CAB Int).

[B75] Steinhauer D. A. Domingo E. Holland J. J. (1992). Lack of evidence for proofreading mechanisms associated with an RNA virus polymerase. Gene 122 (2), 281–288. 10.1016/0378-1119(92)90216-c 1336756

[B76] Sun B.-J. Sun L.-Y. Tugume A. Adams M. Yang J. Xie L.-H. (2013). Selection pressure and founder effects constrain genetic variation in differentiated populations of soilborne bymovirus Wheat yellow mosaic virus (*Potyviridae*) in China. Phytopathology 103 (9), 949–959. 10.1094/phyto-01-13-0013-r 23550972

[B77] Tan Z. Gibbs A. J. Tomitaka Y. Sanchez F. Ponz F. Ohshima K. (2005). Mutations in Turnip mosaic virus genomes that have adapted to Raphanus sativus. J. Gen. Virol. 86 (Pt 2), 501–510. 10.1099/vir.0.80540-0 15659771

[B78] Tatineni S. Elowsky C. Graybosch R. A. (2017). Wheat streak mosaic virus coat protein deletion mutants elicit more severe symptoms than wild-type virus in multiple cereal hosts. Mol. Plant-Microbe Interact. 30 (12), 974–983. 10.1094/MPMI-07-17-0182-R 28840785

[B79] Tatineni S. Robertson C. J. Garnsey S. M. Dawson W. O. (2011). A plant virus evolved by acquiring multiple nonconserved genes to extend its host range. Proc. Natl. Acad. Sci. 108 (42), 17366. 10.1073/pnas.1113227108 21987809 PMC3198328

[B80] Tokuriki N. Oldfield C. J. Uversky V. N. Berezovsky I. N. Tawfik D. S. (2009). Do viral proteins possess unique biophysical features? Trends Biochem. Sci. 34 (2), 53–59. 10.1016/j.tibs.2008.10.009 19062293

[B81] Torres-Barcelo C. Martin S. Daros J. A. Elena S. F. (2008). From hypo- to hypersuppression: effect of amino acid substitutions on the RNA-silencing suppressor activity of the Tobacco etch potyvirus HC-Pro. Genetics 180 (2), 1039–1049. 10.1534/genetics.108.091363 18780745 PMC2567354

[B82] Valli A. Lopez-Moya J. J. Garcia J. A. (2007). Recombination and gene duplication in the evolutionary diversification of P1 proteins in the family Potyviridae. J. Gen. Virol. 88 (Pt 3), 1016–1028. 10.1099/vir.0.82402-0 17325376

[B83] Vijayapalani P. Maeshima M. Nagasaki-Takekuchi N. Miller W. A. (2012). Interaction of the trans-frame potyvirus protein P3N-PIPO with host protein PCaP1 facilitates potyvirus movement. PloS Pathog. 8 (4), e1002639. 10.1371/journal.ppat.1002639 22511869 PMC3325209

[B84] Wamaitha M. J. Nigam D. Maina S. Stomeo F. Wangai A. Njuguna J. N. (2018). Metagenomic analysis of viruses associated with maize lethal necrosis in Kenya. Virol. J. 15 (1), 90. 10.1186/s12985-018-0999-2 29792207 PMC5966901

[B85] Wan J. Basu K. Mui J. Vali H. Zheng H. Laliberte J. F. (2015). Ultrastructural characterization of turnip mosaic virus-induced cellular rearrangements reveals membrane-bound viral particles accumulating in vacuoles. J. Virol. 89 (24), 12441–12456. 10.1128/JVI.02138-15 26423955 PMC4665257

[B86] Wang H. Huang L. F. Cooper J. I. (2006). Analyses on mutation patterns, detection of population bottlenecks, and suggestion of deleterious-compensatory evolution among members of the genus Potyvirus. Arch. Virol. 151 (8), 1625–1633. 10.1007/s00705-006-0741-z 16538419

[B87] Wei T. Zhang C. Hong J. Xiong R. Kasschau K. D. Zhou X. (2010). Formation of complexes at plasmodesmata for potyvirus intercellular movement is mediated by the viral protein P3N-PIPO. PloS Pathog. 6 (6), e1000962. 10.1371/journal.ppat.1000962 20585568 PMC2891837

[B88] Wen R. H. Maroof M. S. Hajimorad M. (2011). Amino acid changes in P3, and not the overlapping pipo-encoded protein, determine virulence of Soybean mosaic virus on functionally immune Rsv1-genotype soybean. Mol. Plant Pathol. 12 (8), 799–807. 10.1111/j.1364-3703.2011.00714.x 21726381 PMC6640218

[B89] White K. A. (2015). The polymerase slips and PIPO exists. EMBO Rep. 16, 885–886. 10.15252/embr.201540871 26160653 PMC4552478

[B90] Wickham H. (2009). ggplot2: Elegant graphics for data analysis. New York: Springer. 10.1007/978-0-387-98141-3

[B91] Wickham H. Chang W. Wickham M. H. (2013). Package 'ggplot2.

[B92] Willemsen A. Zwart M. P. Tromas N. Majer E. Daros J. A. Elena S. F. (2016). Multiple Barriers to the Evolution of Alternative Gene Orders in a Positive-Strand RNA Virus. Genetics 202 (4), 1503–1521. 10.1534/genetics.115.185017 26868766 PMC4905534

[B93] Wong E. H. Smith D. K. Rabadan R. Peiris M. Poon L. L. (2010). Codon usage bias and the evolution of influenza A viruses. Codon Usage Biases of Influenza Virus. BMC evolutionary Biol. 10 (1), 253. 10.1186/1471-2148-10-253 PMC2933640 20723216

[B94] Wylie S. J. Adams M. Chalam C. Kreuze J. Lopez-Moya J. J. Ohshima K. (2017). ICTV Virus Taxonomy Profile: Potyviridae. J. Gen. Virol. 98 (3), 352–354. 10.1099/jgv.0.000740 28366187 PMC5797945

[B95] Wylie S. J. Kueh J. Welsh B. Smith L. J. Jones M. G. K. Jones R. A. C. (2002). A non-aphid-transmissible isolate of bean yellow mosaic potyvirus has an altered NAG motif in its coat protein. Arch. Virol. 147 (9), 1813–1820. 10.1007/s00705-002-0846-y 12209319

[B96] Zamora M. Méndez-López E. Agirrezabala X. Cuesta R. Lavín J. L. Sánchez-Pina M. A. (2017). Potyvirus virion structure shows conserved protein fold and RNA binding site in ssRNA viruses. Sci. Adv. 3 (9), eaao2182–eaao2182. 10.1126/sciadv.aao2182 28948231 PMC5606705

[B97] Zhang J. Wang M. Liu W.-q. Zhou J.-H. Chen H.t. Ma L.-n. (2011). Analysis of codon usage and nucleotide composition bias in polioviruses. Virol. J. 8, 146. 10.1186/1743-422x-8-146 21450075 PMC3079669

[B98] Zhao H. Li Q. Li J. Zeng C. Hu S. Yu J. (2006). The study of neighboring nucleotide composition and transition/transversion bias. Sci. China Ser. C: Life Sci. 49 (4), 395–402. 10.1007/s11427-006-2002-5 16989286

