# Supplementary material for: Genome-Wide Variation in Potyviruses
Source: Front Plant Sci. 2019 Nov 12;10:1439. doi: 10.3389/fpls.2019.01439 (PMC6863122; doi:10.3389/fpls.2019.01439)
Supplement: Supplementary Table S1 — Nucleotide and polyprotein features of Potyviruses used in this study. [file Table_1.docx]

**Table 1. Nucleotide and polyprotein features of Potyviruses and other viruses used in this study.** For each virus, as reference we used an accession with the longest sequence, had full length nucleotide and polyprotein sequence, and was annotated. To account for variation length, accessions were required to be at least 95% the length of the reference accession. The total number of accessions available in NCBI and the number of accessions containing at least 95% of the genome or polyprotein is indicated.

| \| \| *Potyviruses* \| Reference \| Nucleotide \| \| \| \| Polyprotein \| \| \| \| \| --- \| --- \| --- \| --- \| --- \| --- \| --- \| --- \| --- \| --- \| \|  \|  \| **Length (nt)** \| **Total Accessions** \| **NT Length as 95%** \| **>= 95%** \| **Length (AA)** \| **Total Accessions** \| **Protein Length as 95%** \| **>= 95%** \| \| Agropyron mosaic virus \| EU410442.1 \| 9842 \| 2* \| 9349.9 \| 5 \| 3174 \| 13 \| 3015.3 \| 5 \| \| Asparagus virus 1 \| NC_025821.1 \| 9741 \| 22 \| 9253.95 \| 3 \| 3112 \| 22 \| 2956.4 \| 3 \| \| Banana bract mosaic virus \| KT456531.1 \| 9713 \| 141 \| 9227.35 \| 4 \| 3125 \| 149 \| 2968.75 \| 4 \| \| Basella rugose mosaic virus \| DQ851494.1 \| 9882 \| 6 \| 9387.9 \| 4 \| 3079 \| 17 \| 2925.05 \| 4 \| \| Bean common mosaic necrosis virus \| AY864314.2 \| 9893 \| 34 \| 9398.35 \| 13 \| 3071 \| 46 \| 2917.45 \| 14 \| \| Bean common mosaic virus \| KC478389.1 \| 10080 \| 361 \| 9576 \| 63 \| 3222 \| 356 \| 3061 \| 63 \| \| Bean yellow mosaic virus \| NC_003492.1 \| 9532 \| 265 \| 9055 \| 48 \| 3056 \| 269 \| 2903 \| 49 \| \| Beet mosaic virus \| NC_005304.1 \| 9591 \| 18 \| 9111.45 \| 5 \| 3058 \| 31 \| 2905.1 \| 6 \| \| Bidens mottle virus \| NC_014325.1 \| 9741 \| 19 \| 9253.95 \| 7 \| 3071 \| 31 \| 2917.45 \| 7 \| \| Bean common mosaic virus strain Blackeye \| AY575773.1 \| 9992 \| 18 \| 9492.4 \| 1* \| 3202 \| 15 \| 3041.9 \| 1* \| \| Blue squill virus A \| JQ807999.1 \| 9842 \| 4 \| 9349.9 \| 3 \| 3087 \| 16 \| 2932.65 \| 3 \| \| Calla lily latent virus \| EF105297.1 \| 9731 \| 10 \| 9244.45 \| 4 \| 3104 \| 11 \| 2948.8 \| 4 \| \| Canna yellow streak virus \| NC_013261.1 \| 9502 \| 26 \| 9026.9 \| 11 \| 3040 \| 38 \| 2888 \| 11 \| \| Carrot thin leaf virus \| NC_025254.1 \| 9491 \| 4 \| 9016.45 \| 3 \| 3066 \| 5 \| 2912.7 \| 3 \| \| Catharanthus mosaic virus \| KP742991.1 \| 9636 \| 4 \| 9154.2 \| 3 \| 3054 \| 6 \| 2901.3 \| 3 \| \| Celery mosaic virus \| MF962880.1 \| 10000 \| 11 \| 9500 \| 3 \| 3181 \| 23 \| 3021.95 \| 3 \| \| Chilli veinal mottle virus \| NC_005778.1 \| 9711 \| 134 \| 9225 \| 14 \| 3088 \| 141 \| 2934 \| 12 \| \| Chilli ringspot virus \| KX258620.1 \| 9652 \| 13 \| 9169.4 \| 5 \| 3086 \| 25 \| 2931.7 \| 5 \| \| Clover yellow vein virus \| KU922565.1 \| 9585 \| 46 \| 9105.75 \| 8 \| 3072 \| 58 \| 2918.4 \| 7 \| \| Cocksfoot streak virus \| AF499738.1 \| 9663 \| 21 \| 9179.85 \| 3 \| 3089 \| 32 \| 2934.55 \| 3 \| \| Cowpea aphid-borne mosaic virus \| X00206.1 \| 5889 \| 33 \| 5594.55 \| 2* \| 1866 \| 46 \| 1772.7 \| 3* \| \| Cucurbit Vein Banding Virus \| KY657266.1 \| 9969 \| 3 \| 9470.55 \| 3 \| 3168 \| 17 \| 3009.6 \| 3 \| \| Cyrtanthus elatus virus A \| KX575832.1 \| 9942 \| 6 \| 9444.9 \| 3 \| 3102 \| 18 \| 2946.9 \| 3 \| \| Daphne mosaic virus \| DQ299908.1 \| 9548 \| 3 \| 9070.6 \| 2* \| 3071 \| 14 \| 2917.45 \| 2* \| \| Dasheen mosaic virus \| AJ298033.1 \| 10038 \| 82 \| 9536.1 \| 7 \| 3190 \| 93 \| 3030.5 \| 7 \| \| East asian passiflora virus \| KY614052.1 \| 10169 \| 56 \| 9660.55 \| 26 \| 3224 \| 67 \| 3062.8 \| 26 \| \| Freesia mosaic virus \| GU214748.1 \| 9489 \| 8 \| 9014.55 \| 3 \| 3078 \| 20 \| 2924.1 \| 3 \| \| Fritillary virus Y \| AM039800.1 \| 9656 \| 2* \| 9173.2 \| 2* \| 3078 \| 13 \| 2924.1 \| 2* \| \| Habenaria mosaic virus \| NC_021786.1 \| 9499 \| 3 \| 9024.05 \| 3 \| 3054 \| 16 \| 2901.3 \| 3 \| \| Hardenbergia mosaic virus \| HQ161081.1 \| 9682 \| 47 \| 9197.9 \| 9 \| 3082 \| 59 \| 2927.9 \| 9 \| \| Hippeastrum mosaic virus \| JQ723474.1 \| 9660 \| 12 \| 9177 \| 3 \| 3156 \| 24 \| 2998.2 \| 3 \| \| Iranian johnsongrass mosaic virus \| KT899778.1 \| 9544 \| 16 \| 9066.8 \| 3 \| 3054 \| 17 \| 2901.3 \| 3 \| \| Japanese yam mosaic virus \| NC_000947.1 \| 9760 \| 41 \| 9272 \| 8 \| 3134 \| 51 \| 2977.3 \| 9 \| \| Jasmine virus T \| KX398054.1 \| 9660 \| 5 \| 9177 \| 4 \| 3073 \| 13 \| 2919.35 \| 4 \| \| Johnsongrass mosaic virus \| KT289893.1 \| 9874 \| 37 \| 9380.3 \| 5 \| 3070 \| 51 \| 2916.5 \| 5 \| \| Konjac mosaic virus \| AB219545.1 \| 9544 \| 19 \| 9066.8 \| 2* \| 3088 \| 28 \| 2933.6 \| 2* \| \| Leek yellow stripe virus \| NC_004011.1 \| 10142 \| 187 \| 96349 \| 17 \| 3152 \| 189 \| 2995 \| 13 \| \| Lettuce mosaic virus \| AJ278854.1 \| 10080 \| 288 \| 9576 \| 32 \| 3255 \| 305 \| 3092.25 \| 32 \| \| Lily mottle virus \| AM048875.1 \| 9648 \| 108 \| 9165.6 \| 9 \| 3096 \| 115 \| 2941.2 \| 7 \| \| Maize dwarf mosaic virus \| FM883164.2 \| 9554 \| 238 \| 9076.3 \| 9 \| 3054 \| 246 \| 2901.3 \| 9 \| \| Moroccan watermelon mosaic virus \| EF579955.1 \| 9730 \| 25 \| 9243.5 \| 5 \| 3124 \| 38 \| 2967.8 \| 5 \| \| Narcissus degeneration virus \| AM182028.1 \| 9816 \| 9 \| 9325.2 \| 3 \| 3184 \| 20 \| 3024.8 \| 3 \| \| Narcissus late season yellows virus \| NC_023628.1 \| 9687 \| 20 \| 9202.65 \| 4 \| 3098 \| 30 \| 2943.1 \| 4 \| \| Narcissus yellow stripe virus \| NC_011541.1 \| 9650 \| 11 \| 9168 \| 5 \| 3103 \| 22 \| 2948 \| 5 \| \| Onion yellow dwarf virus \| NC_005029.1 \| 10538 \| 224 \| 10011 \| 10 \| 3403 \| 209 \| 3232.85 \| 10 \| \| Ornithogalum mosaic virus \| JQ807996.1 \| 9447 \| 40 \| 8974.65 \| 6 \| 3015 \| 53 \| 2864.25 \| 6 \| \| Papaya leaf distortion mosaic virus \| NC_005028.1 \| 10155 \| 17 \| 9647.25 \| 7 \| 3269 \| 28 \| 3105.55 \| 7 \| \| Papaya ringspot virus \| KP462721.1 \| 10349 \| 1130 \| 9831.55 \| 55 \| 3344 \| 1073 \| 3177 \| 56 \| \| Passion fruit woodiness virus \| KX577780.1 \| 9689 \| 29 \| 9204.55 \| 4 \| 3086 \| 42 \| 2931.7 \| 4 \| \| Pea seed-borne mosaic virus \| KU870637.1 \| 9939 \| 66 \| 9442.05 \| 5 \| 3206 \| 77 \| 3045.7 \| 6 \| \| Peace lily mosaic virus \| DQ851494.1 \| 9882 \| 1 \| 9387.9 \| 1* \| 3079 \| 1 \| 2925.05 \| 1* \| \| Peanut mottle virus \| KU708532.1 \| 9734 \| 23 \| 9247.3 \| 6 \| 3099 \| 36 \| 2944.05 \| 7 \| \| Peanut stripe virus (Bean common mosaic virus strain peanut stripe) \| U34972.1 \| 10086 \| 116 \| 9581.7 \| 5 \| 3222 \| 117 \| 3060.9 \| 5 \| \| Pennisetum mosaic virus \| JX070139.1 \| 9613 \| 34 \| 9132.35 \| 23 \| 3065 \| 45 \| 2911.75 \| 23 \| \| Pepper mottle virus \| AF501591.1 \| 9717 \| 64 \| 9231.15 \| 24 \| 3068 \| 75 \| 2914.6 \| 22 \| \| Pepper severe mosaic virus \| AM181350.1 \| 9890 \| 4 \| 9395.5 \| 2* \| 3077 \| 16 \| 2923.15 \| 2* \| \| Pepper veinal mottle virus \| FJ617225.2 \| 9812 \| 23 \| 9321.4 \| 5 \| 3074 \| 32 \| 2920.3 \| 4 \| \| Peru tomato mosaic virus \| AJ437280.1 \| 9899 \| 18 \| 9404.05 \| 3 \| 3065 \| 28 \| 2911.75 \| 3 \| \| Plum pox virus \| MF370984.1 \| 9975 \| 1498 \| 9476.25 \| 159 \| 3140 \| 1507 \| 2983 \| 158 \| \| Pokeweed mosaic virus \| KU133475.1 \| 9527 \| 6 \| 9050.65 \| 6 \| 3056 \| 17 \| 2903.2 \| 6 \| \| Potato virus A \| AJ131403.1 \| 9605 \| 96 \| 9124.75 \| 15 \| 3059 \| 105 \| 2906.05 \| 16 \| \| Potato virus V \| KP849483.1 \| 9904 \| 64 \| 9408.8 \| 6 \| 3066 \| 62 \| 2912.7 \| 6 \| \| Potato virus Y \| MG591487.1 \| 9724 \| 2880 \| 9238 \| 449 \| 3061 \| 2845 \| 2908 \| 429 \| \| Potato virus Y strain NTN \| AJ890347.1 \| 9706 \| 35 \| 9220.7 \| 13 \| 3062 \| 36 \| 2908.9 \| 13 \| \| Scallion mosaic virus \| AJ316084.1 \| 9324 \| 5 \| 8857.8 \| 2* \| 3001 \| 16 \| 2850.95 \| 2* \| \| Shallot yellow stripe virus \| AJ865076.1 \| 10429 \| 25 \| 9907.55 \| 3 \| 3401 \| 39 \| 3230.95 \| 6 \| \| Sorghum mosaic virus \| KM025054.1 \| 9652 \| 417 \| 9169.4 \| 22 \| 3079 \| 421 \| 2925.05 \| 22 \| \| Soybean mosaic virus \| KY986929.1 \| 9584 \| 494 \| 9105 \| 101 \| 3066 \| 577 \| 2913 \| 123 \| \| Sudan watermelon mosaic virus \| KY623505.1 \| 10232 \| 41 \| 9720.4 \| 2* \| 3295 \| 42 \| 3130.25 \| 2* \| \| Sugarcane mosaic virus \| JX188385.1 \| 9613 \| 1058 \| 9132 \| 91 \| 3077 \| 1037 \| 2923 \| 91 \| \| Sunflower chlorotic mottle virus \| GU181199. \| 9965 \| 9 \| 9466.75 \| 5 \| 3187 \| 21 \| 3027.65 \| 5 \| \| Sweet potato feathery mottle virus \| FJ155666.1 \| 11004 \| 344 \| 10453.8 \| 28 \| 3553 \| 365 \| 3375.35 \| 28 \| \| Sweet potato latent virus \| KP115613.1 \| 10081 \| 60 \| 9576.95 \| 6 \| 3247 \| 61 \| 3084.65 \| 6 \| \| Sweet potato virus 2 \| KX017447.1 \| 10732 \| 16 \| 10195.4 \| 11 \| 3466 \| 30 \| 3292.7 \| 11 \| \| Sweet potato virus C \| MF572064.1 \| 10832 \| 79 \| 10290.4 \| 22 \| 3481 \| 93 \| 3306.95 \| 22 \| \| Sweet potato virus G \| NC_018093.1 \| 10798 \| 132 \| 10258.1 \| 12 \| 3488 \| 142 \| 3313.6 \| 12 \| \| Tamarillo leaf malformation virus \| NC_026615.1 \| 9720 \| 24 \| 9234 \| 3 \| 3073 \| 32 \| 2919.35 \| 3 \| \| Telosma mosaic virus \| DQ851493.1 \| 9689 \| 13 \| 9204.55 \| 2* \| 3082 \| 24 \| 2927.9 \| 2* \| \| Thunberg fritillary mosaic virus \| AJ851866.1 \| 9723 \| 5 \| 9236.85 \| 3 \| 3129 \| 16 \| 2972.55 \| 3 \| \| Tobacco etch virus \| DQ986288 \| 9539 \| 181 \| 9062.05 \| 10 \| 3054 \| 175 \| 2901.3 \| 12 \| \| Tobacco vein banding mosaic virus \| KY499622.1 \| 9596 \| 159 \| 9116.2 \| 18 \| 3079 \| 165 \| 2925.05 \| 15 \| \| Tobacco vein mottling virus \| U38621.1 \| 9475 \| 12 \| 9001.25 \| 3 \| 3023 \| 27 \| 2871.85 \| 4 \| \| Tomato necrotic stunt virus \| NC_017824.1 \| 10057 \| 3 \| 9554.15 \| 3 \| 3132 \| 15 \| 2975.4 \| 3 \| \| Turnip mosaic virus \| AF169561.2 \| 9837 \| 1667 \| 9345 \| 483 \| 3164 \| 1658 \| 3005.8 \| 485 \| \| Verbena virus y \| EU564817.1 \| 9742 \| 2* \| 9254.9 \| 2* \| 3105 \| 13 \| 2949.75 \| 2* \| \| Watermelon mosaic virus \| KT992083.1 \| 10145 \| 691 \| 9637.75 \| 86 \| 3225 \| 702 \| 3063.75 \| 86 \| \| White Lupin mosaic virus \| DQ641248.1 \| 9512 \| 1* \| 9036.4 \| 1* \| 3056 \| 1* \| 2903.2 \| 1* \| \| Wild potato mosaic virus \| AJ437279.1 \| 9878 \| 2* \| 9384.1 \| 2* \| 3065 \| 12 \| 2911.75 \| 2* \| \| Wild tomato mosaic virus \| KM401435.1 \| 9696 \| 12 \| 9211.2 \| 3 \| 3075 \| 23 \| 2921.25 \| 3 \| \| Wisteria vein mosaic virus \| AY656816.1 \| 9695 \| 12 \| 9210.25 \| 2* \| 3092 \| 22 \| 2937.4 \| 2* \| \| Yam mild mosaic virus \| NC_019412.1 \| 9538 \| 115 \| 9061.1 \| 13 \| 3084 \| 136 \| 2929.8 \| 13 \| \| Yam mosaic virus \| YMU42596 \| 9608 \| 77 \| 9127.6 \| 3 \| 3103 \| 95 \| 2947.85 \| 1* \| \| Zantedeschia mild mosaic virus \| AY626825.4 \| 9973 \| 10 \| 9474.35 \| 3 \| 3176 \| 21 \| 3017.2 \| 3 \| \| Zucchini tigre mosaic virus \| KC345608.1 \| 10346 \| 35 \| 9828.7 \| 5 \| 3349 \| 45 \| 3181.55 \| 5 \| \| Zucchini yellow mosaic virus \| KX499498.1 \| 9592 \| 1190 \| 9112 \| 90 \| 3080 \| 1152 \| 2926 \| 87 \| \| Average \|  \| 9,799 \|  \|  \|  \| 3,125 \|  \|  \|  \| \| Maize chlorotic mottle virus ^1^ \| X14736.2 \| 4437 \| 95 \| 53 \| 236^2^ \|  \|  \|  \|  \| \| \| --- \| --- \| --- \| --- \| --- \| --- \| --- \| --- \| --- \| --- \| --- \| --- \| --- \| --- \| --- \| --- \| --- \| --- \| --- \| --- \| --- \| --- \| --- \| --- \| --- \| --- \| --- \| --- \| --- \| --- \| --- \| --- \| --- \| --- \| --- \| --- \| --- \| --- \| --- \| --- \| --- \| --- \| --- \| --- \| --- \| --- \| --- \| --- \| --- \| --- \| --- \| --- \| --- \| --- \| --- \| --- \| --- \| --- \| --- \| --- \| --- \| --- \| --- \| --- \| --- \| --- \| --- \| --- \| --- \| --- \| --- \| --- \| --- \| --- \| --- \| --- \| --- \| --- \| --- \| --- \| --- \| --- \| --- \| --- \| --- \| --- \| --- \| --- \| --- \| --- \| --- \| --- \| --- \| --- \| --- \| --- \| --- \| --- \| --- \| --- \| --- \| --- \| --- \| --- \| --- \| --- \| --- \| --- \| --- \| --- \| --- \| --- \| --- \| --- \| --- \| --- \| --- \| --- \| --- \| --- \| --- \| --- \| --- \| --- \| --- \| --- \| --- \| --- \| --- \| --- \| --- \| --- \| --- \| --- \| --- \| --- \| --- \| --- \| --- \| --- \| --- \| --- \| --- \| --- \| --- \| --- \| --- \| --- \| --- \| --- \| --- \| --- \| --- \| --- \| --- \| --- \| --- \| --- \| --- \| --- \| --- \| --- \| --- \| --- \| --- \| --- \| --- \| --- \| --- \| --- \| --- \| --- \| --- \| --- \| --- \| --- \| --- \| --- \| --- \| --- \| --- \| --- \| --- \| --- \| --- \| --- \| --- \| --- \| --- \| --- \| --- \| --- \| --- \| --- \| --- \| --- \| --- \| --- \| --- \| --- \| --- \| --- \| --- \| --- \| --- \| --- \| --- \| --- \| --- \| --- \| --- \| --- \| --- \| --- \| --- \| --- \| --- \| --- \| --- \| --- \| --- \| --- \| --- \| --- \| --- \| --- \| --- \| --- \| --- \| --- \| --- \| --- \| --- \| --- \| --- \| --- \| --- \| --- \| --- \| --- \| --- \| --- \| --- \| --- \| --- \| --- \| --- \| --- \| --- \| --- \| --- \| --- \| --- \| --- \| --- \| --- \| --- \| --- \| --- \| --- \| --- \| --- \| --- \| --- \| --- \| --- \| --- \| --- \| --- \| --- \| --- \| --- \| --- \| --- \| --- \| --- \| --- \| --- \| --- \| --- \| --- \| --- \| --- \| --- \| --- \| --- \| --- \| --- \| --- \| --- \| --- \| --- \| --- \| --- \| --- \| --- \| --- \| --- \| --- \| --- \| --- \| --- \| --- \| --- \| --- \| --- \| --- \| --- \| --- \| --- \| --- \| --- \| --- \| --- \| --- \| --- \| --- \| --- \| --- \| --- \| --- \| --- \| --- \| --- \| --- \| --- \| --- \| --- \| --- \| --- \| --- \| --- \| --- \| --- \| --- \| --- \| --- \| --- \| --- \| --- \| --- \| --- \| --- \| --- \| --- \| --- \| --- \| --- \| --- \| --- \| --- \| --- \| --- \| --- \| --- \| --- \| --- \| --- \| --- \| --- \| --- \| --- \| --- \| --- \| --- \| --- \| --- \| --- \| --- \| --- \| --- \| --- \| --- \| --- \| --- \| --- \| --- \| --- \| --- \| --- \| --- \| --- \| --- \| --- \| --- \| --- \| --- \| --- \| --- \| --- \| --- \| --- \| --- \| --- \| --- \| --- \| --- \| --- \| --- \| --- \| --- \| --- \| --- \| --- \| --- \| --- \| --- \| --- \| --- \| --- \| --- \| --- \| --- \| --- \| --- \| --- \| --- \| --- \| --- \| --- \| --- \| --- \| --- \| --- \| --- \| --- \| --- \| --- \| --- \| --- \| --- \| --- \| --- \| --- \| --- \| --- \| --- \| --- \| --- \| --- \| --- \| --- \| --- \| --- \| --- \| --- \| --- \| --- \| --- \| --- \| --- \| --- \| --- \| --- \| --- \| --- \| --- \| --- \| --- \| --- \| --- \| --- \| --- \| --- \| --- \| --- \| --- \| --- \| --- \| --- \| --- \| --- \| --- \| --- \| --- \| --- \| --- \| --- \| --- \| --- \| --- \| --- \| --- \| --- \| --- \| --- \| --- \| --- \| --- \| --- \| --- \| --- \| --- \| --- \| --- \| --- \| --- \| --- \| --- \| --- \| --- \| --- \| --- \| --- \| --- \| --- \| --- \| --- \| --- \| --- \| --- \| --- \| --- \| --- \| --- \| --- \| --- \| --- \| --- \| --- \| --- \| --- \| --- \| --- \| --- \| --- \| --- \| --- \| --- \| --- \| --- \| --- \| --- \| --- \| --- \| --- \| --- \| --- \| --- \| --- \| --- \| --- \| --- \| --- \| --- \| --- \| --- \| --- \| --- \| --- \| --- \| --- \| --- \| --- \| --- \| --- \| --- \| --- \| --- \| --- \| --- \| --- \| --- \| --- \| --- \| --- \| --- \| --- \| --- \| --- \| --- \| --- \| --- \| --- \| --- \| --- \| --- \| --- \| --- \| --- \| --- \| --- \| --- \| --- \| --- \| --- \| --- \| --- \| --- \| --- \| --- \| --- \| --- \| --- \| --- \| --- \| --- \| --- \| --- \| --- \| --- \| --- \| --- \| --- \| --- \| --- \| --- \| --- \| --- \| --- \| --- \| --- \| --- \| --- \| --- \| --- \| --- \| --- \| --- \| --- \| --- \| --- \| --- \| --- \| --- \| --- \| --- \| --- \| --- \| --- \| --- \| --- \| --- \| --- \| --- \| --- \| --- \| --- \| --- \| --- \| --- \| --- \| --- \| --- \| --- \| --- \| --- \| --- \| --- \| --- \| --- \| --- \| --- \| --- \| --- \| --- \| --- \| --- \| --- \| --- \| --- \| --- \| --- \| --- \| --- \| --- \| --- \| --- \| --- \| --- \| --- \| --- \| --- \| --- \| --- \| --- \| --- \| --- \| --- \| --- \| --- \| --- \| --- \| --- \| --- \| --- \| --- \| --- \| --- \| --- \| --- \| --- \| --- \| --- \| --- \| --- \| --- \| --- \| --- \| --- \| --- \| --- \| --- \| --- \| --- \| --- \| --- \| --- \| --- \| --- \| --- \| --- \| --- \| --- \| --- \| --- \| --- \| --- \| --- \| --- \| --- \| --- \| --- \| --- \| --- \| --- \| --- \| --- \| --- \| --- \| --- \| --- \| --- \| --- \| --- \| --- \| --- \| --- \| --- \| --- \| --- \| --- \| --- \| --- \| --- \| --- \| --- \| --- \| --- \| --- \| --- \| --- \| --- \| --- \| --- \| --- \| --- \| --- \| --- \| --- \| --- \| --- \| --- \| --- \| --- \| --- \| --- \| --- \| --- \| --- \| --- \| --- \| --- \| --- \| --- \| --- \| --- \| --- \| --- \| --- \| --- \| --- \| --- \| --- \| --- \| --- \| --- \| --- \| --- \| --- \| --- \| --- \| --- \| --- \| --- \| --- \| --- \| --- \| --- \| --- \| --- \| --- \| --- \| --- \| --- \| --- \| --- \| --- \| --- \| --- \| --- \| --- \| --- \| --- \| --- \| --- \| --- \| --- \| --- \| --- \| --- \| --- \| --- \| --- \| --- \| --- \| --- \| --- \| --- \| --- \| --- \| --- \| --- \| --- \| --- \| --- \| --- \| --- \| --- \| --- \| --- \| --- \| --- \| --- \| --- \| --- \| --- \| --- \| --- \| --- \| --- \| --- \| --- \| --- \| --- \| --- \| --- \| --- \| --- \| --- \| --- \| --- \| --- \| --- \| --- \| --- \| --- \| --- \| --- \| --- \| --- \| --- \| --- \| --- \| --- \| --- \| --- \| --- \| --- \| --- \| --- \| --- \| --- \| --- \| --- \| --- \| --- \| --- \| --- \| --- \| --- \| --- \| --- \| --- \| --- \| --- \| --- \| --- \| --- \| --- \| --- \| --- \| --- \| --- \| --- \| --- \| --- \| --- \| --- \| --- \| --- \| --- \| --- \| --- \| --- \| --- \| --- \| --- \| --- \| --- \| --- \| --- \| --- \| --- \| --- \| --- \| --- \| --- \| --- \| --- \| --- \| --- \| --- \| --- \| --- \| --- \| --- \| --- \| --- \| --- \| --- \| --- \| --- \| --- \| --- \| --- \| --- \| --- \| --- \| --- \| --- \| --- \| --- \| --- \| --- \| --- \| --- \| --- \| --- \| --- \| --- \| --- \| --- \| --- \| --- \| --- \| --- \| --- \| --- \| --- \| --- \| --- \| --- \| --- \| --- \| --- \| --- \| --- \| --- \| --- \| --- \| --- \| --- \| --- \| --- \| --- \| --- \| --- \| --- \| --- \| --- \| |
| --- | --- | --- | --- | --- | --- | --- | --- | --- | --- | --- | --- | --- | --- | --- | --- | --- | --- | --- | --- | --- | --- | --- | --- | --- | --- | --- | --- | --- | --- | --- | --- | --- | --- | --- | --- | --- | --- | --- | --- | --- | --- | --- | --- | --- | --- | --- | --- | --- | --- | --- | --- | --- | --- | --- | --- | --- | --- | --- | --- | --- | --- | --- | --- | --- | --- | --- | --- | --- | --- | --- | --- | --- | --- | --- | --- | --- | --- | --- | --- | --- | --- | --- | --- | --- | --- | --- | --- | --- | --- | --- | --- | --- | --- | --- | --- | --- | --- | --- | --- | --- | --- | --- | --- | --- | --- | --- | --- | --- | --- | --- | --- | --- | --- | --- | --- | --- | --- | --- | --- | --- | --- | --- | --- | --- | --- | --- | --- | --- | --- | --- | --- | --- | --- | --- | --- | --- | --- | --- | --- | --- | --- | --- | --- | --- | --- | --- | --- | --- | --- | --- | --- | --- | --- | --- | --- | --- | --- | --- | --- | --- | --- | --- | --- | --- | --- | --- | --- | --- | --- | --- | --- | --- | --- | --- | --- | --- | --- | --- | --- | --- | --- | --- | --- | --- | --- | --- | --- | --- | --- | --- | --- | --- | --- | --- | --- | --- | --- | --- | --- | --- | --- | --- | --- | --- | --- | --- | --- | --- | --- | --- | --- | --- | --- | --- | --- | --- | --- | --- | --- | --- | --- | --- | --- | --- | --- | --- | --- | --- | --- | --- | --- | --- | --- | --- | --- | --- | --- | --- | --- | --- | --- | --- | --- | --- | --- | --- | --- | --- | --- | --- | --- | --- | --- | --- | --- | --- | --- | --- | --- | --- | --- | --- | --- | --- | --- | --- | --- | --- | --- | --- | --- | --- | --- | --- | --- | --- | --- | --- | --- | --- | --- | --- | --- | --- | --- | --- | --- | --- | --- | --- | --- | --- | --- | --- | --- | --- | --- | --- | --- | --- | --- | --- | --- | --- | --- | --- | --- | --- | --- | --- | --- | --- | --- | --- | --- | --- | --- | --- | --- | --- | --- | --- | --- | --- | --- | --- | --- | --- | --- | --- | --- | --- | --- | --- | --- | --- | --- | --- | --- | --- | --- | --- | --- | --- | --- | --- | --- | --- | --- | --- | --- | --- | --- | --- | --- | --- | --- | --- | --- | --- | --- | --- | --- | --- | --- | --- | --- | --- | --- | --- | --- | --- | --- | --- | --- | --- | --- | --- | --- | --- | --- | --- | --- | --- | --- | --- | --- | --- | --- | --- | --- | --- | --- | --- | --- | --- | --- | --- | --- | --- | --- | --- | --- | --- | --- | --- | --- | --- | --- | --- | --- | --- | --- | --- | --- | --- | --- | --- | --- | --- | --- | --- | --- | --- | --- | --- | --- | --- | --- | --- | --- | --- | --- | --- | --- | --- | --- | --- | --- | --- | --- | --- | --- | --- | --- | --- | --- | --- | --- | --- | --- | --- | --- | --- | --- | --- | --- | --- | --- | --- | --- | --- | --- | --- | --- | --- | --- | --- | --- | --- | --- | --- | --- | --- | --- | --- | --- | --- | --- | --- | --- | --- | --- | --- | --- | --- | --- | --- | --- | --- | --- | --- | --- | --- | --- | --- | --- | --- | --- | --- | --- | --- | --- | --- | --- | --- | --- | --- | --- | --- | --- | --- | --- | --- | --- | --- | --- | --- | --- | --- | --- | --- | --- | --- | --- | --- | --- | --- | --- | --- | --- | --- | --- | --- | --- | --- | --- | --- | --- | --- | --- | --- | --- | --- | --- | --- | --- | --- | --- | --- | --- | --- | --- | --- | --- | --- | --- | --- | --- | --- | --- | --- | --- | --- | --- | --- | --- | --- | --- | --- | --- | --- | --- | --- | --- | --- | --- | --- | --- | --- | --- | --- | --- | --- | --- | --- | --- | --- | --- | --- | --- | --- | --- | --- | --- | --- | --- | --- | --- | --- | --- | --- | --- | --- | --- | --- | --- | --- | --- | --- | --- | --- | --- | --- | --- | --- | --- | --- | --- | --- | --- | --- | --- | --- | --- | --- | --- | --- | --- | --- | --- | --- | --- | --- | --- | --- | --- | --- | --- | --- | --- | --- | --- | --- | --- | --- | --- | --- | --- | --- | --- | --- | --- | --- | --- | --- | --- | --- | --- | --- | --- | --- | --- | --- | --- | --- | --- | --- | --- | --- | --- | --- | --- | --- | --- | --- | --- | --- | --- | --- | --- | --- | --- | --- | --- | --- | --- | --- | --- | --- | --- | --- | --- | --- | --- | --- | --- | --- | --- | --- | --- | --- | --- | --- | --- | --- | --- | --- | --- | --- | --- | --- | --- | --- | --- | --- | --- | --- | --- | --- | --- | --- | --- | --- | --- | --- | --- | --- | --- | --- | --- | --- | --- | --- | --- | --- | --- | --- | --- | --- | --- | --- | --- | --- | --- | --- | --- | --- | --- | --- | --- | --- | --- | --- | --- | --- | --- | --- | --- | --- | --- | --- | --- | --- | --- | --- | --- | --- | --- | --- | --- | --- | --- | --- | --- | --- | --- | --- | --- | --- | --- | --- | --- | --- | --- | --- | --- | --- | --- | --- | --- | --- | --- | --- | --- | --- | --- | --- | --- | --- | --- | --- | --- | --- | --- | --- | --- | --- | --- | --- | --- | --- | --- | --- | --- | --- | --- | --- | --- | --- | --- | --- | --- | --- | --- | --- | --- | --- | --- | --- | --- | --- | --- | --- | --- | --- | --- | --- | --- | --- | --- | --- | --- | --- | --- | --- | --- | --- | --- | --- | --- | --- | --- | --- | --- | --- | --- | --- | --- | --- | --- | --- | --- | --- | --- | --- | --- | --- | --- | --- | --- | --- | --- | --- | --- | --- | --- | --- | --- | --- | --- | --- | --- | --- | --- | --- | --- | --- | --- | --- | --- | --- | --- | --- | --- | --- | --- | --- | --- | --- | --- | --- | --- | --- | --- | --- | --- | --- | --- | --- | --- | --- | --- | --- | --- | --- | --- | --- | --- | --- | --- | --- | --- | --- | --- | --- | --- | --- | --- | --- | --- | --- | --- | --- | --- | --- | --- | --- | --- | --- | --- | --- | --- | --- | --- | --- | --- | --- | --- | --- | --- | --- | --- | --- | --- | --- | --- | --- | --- | --- | --- | --- | --- | --- | --- | --- | --- | --- | --- | --- | --- | --- | --- | --- | --- | --- | --- | --- | --- | --- | --- | --- | --- | --- | --- | --- | --- | --- | --- | --- | --- |

1. Machomovirus used as control.

2. Coat protein.

* showing the viruses where SNPs couldn’t be identified because of less than 3 number of sequences.
