# Supplementary material for: Genome-Wide Variation in Potyviruses
Source: Front Plant Sci. 2019 Nov 12;10:1439. doi: 10.3389/fpls.2019.01439 (PMC6863122; doi:10.3389/fpls.2019.01439)
Supplement: Supplementary Table S4 — Number of sites under episodic positively selection as reported by SLAC (pervasive) and MEME (episodic) for ten cistrons in a selected group of potyviruses. [file Table_4.docx]

**Table S4A.** Number of sites under episodic positively selection as reported by SLAC (pervasive)

and MEME (episodic) for ten cistrons in a selected group of potyviruses^1,2^.

| **Potyvirus** | **Selection Type** | **P1** | **HC-Pro** | **P3** | **6K1** | **CI** | **6K2** | **VPg** | **NIa** | **NIb** | **CP** |
| --- | --- | --- | --- | --- | --- | --- | --- | --- | --- | --- | --- |
| SCMV | PS | 15 | 5 | 10 | 0 | 3 | 0 | 6 | 1 | 1 | 18 |
|  | ES | 10 | 8 | 4 | 0 | 3 | 0 | 7 | 3 | 2 | 14 |
| PPV | PS | 20 | 3 | 20 | 0 | 5 | 0 | 0 | 11 | 16 | 15 |
|  | ES | 18 | 2 | 17 | 0 | 7 | 0 | 0 | 15 | 10 | 19 |
| BYMV | PS | 16 | 15 | 14 | 0 | 15 | 3 | 4 | 10 | 14 | 17 |
|  | ES | 18 | 13 | 12 | 0 | 8 | 5 | 6 | 12 | 11 | 19 |
| NYSV | PS | 22 | 13 | 19 | 1 | 0 | 1 | 0 | 11 | 7 | 18 |
|  | ES | 17 | 8 | 18 | 3 | 0 | 2 | 0 | 6 | 2 | 10 |
| JYMV | PS | 23 | 14 | 15 | 0 | 0 | 0 | 3 | 0 | 9 | 12 |
|  | ES | 14 | 9 | 13 | 0 | 0 | 0 | 4 | 0 | 8 | 2 |
| CVMV | PS | 9 | 11 | 13 | 0 | 0 | 1 | 4 | 2 | 9 | 11 |
|  | ES | 10 | 6 | 9 | 0 | 0 | 1 | 5 | 2 | 10 | 13 |
| DMV | PS | 8 | 0 | 8 | 1 | 2 | 0 | 1 | 3 | 8 | 11 |
|  | ES | 11 | 0 | 8 | 1 | 3 | 0 | 1 | 5 | 9 | 6 |
| WMV | PS | 19 | 12 | 14 | 0 | 4 | 2 | 7 | 10 | 14 | 11 |
|  | ES | 11 | 9 | 9 | 0 | 6 | 2 | 6 | 11 | 7 | 13 |
| LYSV | PS | 14 | 4 | 8 | 0 | 0 | 0 | 3 | 10 | 13 | 16 |
|  | ES | 13 | 6 | 8 | 0 | 0 | 0 | 4 | 8 | 12 | 6 |
| BCMV | PS | 16 | 8 | 14 | 6 | 10 | 5 | 5 | 7 | 8 | 10 |
|  | ES | 18 | 36 | 35 | 7 | 28 | 4 | 14 | 12 | 27 | 31 |
| SMV | PS | 6 | 22 | 14 | 1 | 2 | 0 | 0 | 0 | 0 | 1 |
|  | ES | 12 | 21 | 11 | 1 | 2 | 0 | 0 | 0 | 0 | 3 |
| PRSV | PS | 30 | 5 | 4 | 0 | 0 | 0 | 1 | 6 | 1 | 5 |
|  | ES | 25 | 1 | 1 | 0 | 0 | 0 | 1 | 7 | 1 | 4 |
| OYDV | PS | 17 | 4 | 7 | 4 | 28 | 0 | 0 | 13 | 4 | 0 |
|  | ES | 10 | 2 | 5 | 2 | 29 | 0 | 0 | 7 | 6 | 0 |
| TuMV | PS | 59 | 5 | 23 | 1 | 5 | 0 | 6 | 5 | 13 | 16 |
|  | ES | 65 | 7 | 16 | 3 | 3 | 0 | 7 | 6 | 12 | 15 |
| ZYMV | PS | 14 | 4 | 5 | 0 | 4 | 0 | 5 | 0 | 14 | 15 |
|  | ES | 12 | 4 | 5 | 0 | 4 | 0 | 3 | 0 | 10 | 10 |
| PVY | PS | 17 | 8 | 15 | 0 | 8 | 0 | 10 | 18 | 16 | 17 |
|  | ES | 16 | 9 | 14 | 0 | 7 | 0 | 10 | 13 | 15 | 15 |

1. p value > 0.05 and posterior probability 0.95.

2. For sites under negative selection and length of the protein, see parts B, and C,

respectively.

**Table S4B.** Number of sites under negative selection as reported by SLAC (pervasive) for ten

cistrons in a selected group of potyviruses^1^.

| **Potyvirus** | **P1** | **HCPro** | **P3** | **6K1** | **CI** | **6K2** | **VPg** | **NIa** | **NIb** | **CP** |
| --- | --- | --- | --- | --- | --- | --- | --- | --- | --- | --- |
| ***SCMV*** | 153 | 403 | 233 | 58 | 568 | 38 | 157 | 202 | 446 | 180 |
| ***PPV*** | 184 | 359 | 200 | 45 | 659 | 36 | 151 | 184 | 395 | 139 |
| ***BYMV*** | 186 | 357 | 189 | 43 | 491 | 39 | 149 | 188 | 275 | 263 |
| ***NYSV*** | 61 | 224 | 125 | 20 | 324 | 19 | 82 | 98 | 182 | 88 |
| ***JYMV*** | 40 | 117 | 85 | 16 | 191 | 11 | 56 | 81 | 166 | 61 |
| ***CVMV*** | 51 | 94 | 45 | 14 | 128 | 16 | 37 | 64 | 112 | 32 |
| ***DMV*** | 183 | 278 | 162 | 26 | 368 | 23 | 78 | 130 | 284 | 116 |
| ***WMV*** | 221 | 397 | 233 | 47 | 559 | 44 | 160 | 205 | 420 | 171 |
| ***LYSV*** | 84 | 223 | 119 | 22 | 316 | 29 | 70 | 99 | 167 | 74 |
| ***BCMV*** | 162 | 307 | 159 | 29 | 425 | 33 | 113 | 161 | 333 | 100 |
| ***SMV*** | 69 | 241 | 182 | 12 | 422 | 32 | 119 | 171 | 342 | 163 |
| ***PRSV*** | 364 | 361 | 246 | 43 | 513 | 49 | 157 | 195 | 157 | 174 |
| ***OYDV*** | 180 | 326 | 283 | 36 | 398 | 40 | 136 | 162 | 288 | 103 |
| ***TuMV*** | 239 | 393 | 242 | 48 | 555 | 45 | 156 | 207 | 385 | 215 |
| ***ZYMV*** | 100 | 238 | 147 | 32 | 352 | 29 | 87 | 130 | 281 | 141 |
| ***PVY*** | 180 | 329 | 300 | 39 | 477 | 28 | 85 | 124 | 273 | 154 |

1. p value > 0.05 and posterior probability 0.95.

**Table S4C.** Length (number of codons) for ten cistrons for a selected group of potyviruses.

| **Potyvirus** | **P1** | **HCPro** | **P3** | **6K1** | **CI** | **6K2** | **VPg** | **NIa** | **NIb** | **CP** |
| --- | --- | --- | --- | --- | --- | --- | --- | --- | --- | --- |
| *SCMV* | 223 | 460 | 347 | 67 | 638 | 53 | 189 | 242 | 522 | 324 |
| *PPV* | 308 | 458 | 350 | 52 | 635 | 53 | 193 | 243 | 518 | 315 |
| *BYMV* | 290 | 457 | 348 | 53 | 635 | 53 | 191 | 243 | 353 | 439 |
| *NYSV* | 317 | 458 | 353 | 52 | 644 | 53 | 191 | 243 | 517 | 274 |
| *JYMV* | 270 | 337 | 259 | 36 | 453 | 36 | 139 | 162 | 343 | 290 |
| *CVMV* | 334 | 424 | 344 | 54 | 641 | 51 | 191 | 242 | 520 | 287 |
| *DMV* | 385 | 458 | 346 | 52 | 634 | 53 | 190 | 243 | 516 | 314 |
| *WMV* | 300 | 458 | 344 | 54 | 641 | 51 | 191 | 242 | 520 | 287 |
| *LYSV* | 362 | 456 | 359 | 52 | 635 | 53 | 192 | 242 | 513 | 288 |
| *BCMV* | 443 | 457 | 347 | 52 | 634 | 53 | 190 | 243 | 516 | 288 |
| *SMV* | 308 | 457 | 347 | 52 | 634 | 53 | 190 | 243 | 517 | 265 |
| *PRSV* | 547 | 457 | 345 | 52 | 635 | 57 | 189 | 238 | 537 | 287 |
| *OYDV* | 458 | 460 | 530 | 51 | 638 | 53 | 196 | 242 | 518 | 259 |
| *TuMV* | 362 | 458 | 355 | 52 | 644 | 52 | 192 | 243 | 517 | 288 |
| *ZYMV* | 310 | 456 | 346 | 52 | 634 | 53 | 190 | 243 | 517 | 279 |
| *PVY* | 283 | 456 | 366 | 51 | 634 | 52 | 188 | 244 | 521 | 268 |
|  |  |  |  |  |  |  |  |  |  |  |
